# The sclerotome is the source of the dorsal and anal fin skeleton and its expansion is required for median fin development

**DOI:** 10.1242/dev.203025

**Published:** 2024-12-13

**Authors:** Raisa Bailon-Zambrano, Margaret K. Keating, Emily C. Sales, Abigail R. Nichols, Grace E. Gustafson, Colette A. Hopkins, Katrinka M. Kocha, Peng Huang, Lindsey Barske, James T. Nichols

**Affiliations:** ^1^ Department of Craniofacial Biology, University of Colorado Anschutz Medical Campus, Aurora, CO 80045, USA; ^2^ Department of Biology, University of Oregon, Eugene, OR 97403, USA; ^3^ Department of Biochemistry and Molecular Biology, Cumming School of Medicine, Alberta Children's Hospital Research Institute, University of Calgary, Calgary, Alberta T2N 1N4, Canada; ^4^ Division of Human Genetics, Cincinnati Children's Hospital Medical Center, Department of Pediatrics, University of Cincinnati College of Medicine, Cincinnati, OH 45229, USA

**Keywords:** Axial skeleton, Dorsal fin, Fins, Sclerotome, Skeleton, Zebrafish

## Abstract

Paired locomotion appendages are hypothesized to have redeployed the developmental program of median appendages, such as the dorsal and anal fins. Compared with paired fins, and limbs, median appendages remain surprisingly understudied. Here, we report that a dominant zebrafish mutant, *smoothback* (*smb*), fails to develop a dorsal fin. Moreover, the anal fin is reduced along the antero-posterior axis, and spine defects develop. Mechanistically, the *smb* mutation is caused by an insertion of a *sox10:Gal4VP16* transgenic construct into a non-coding region. The first step in fin, and limb, induction is aggregation of undifferentiated mesenchyme at the appendage development site. In *smb*, this dorsal fin mesenchyme is absent. Lineage tracing demonstrates the previously unknown developmental origin of the mesenchyme, the sclerotome, which also gives rise to the spine. Strikingly, we find that there is significantly less sclerotome in *smb* than in wild type. Our results give insight into the origin and modularity of understudied median fins, which have changed position, number, size, and even disappeared, across evolutionary time.

## Teaser

**Summary:** The sclerotome, an embryonic structure within the somites, is the source of median fin cells and is reduced in smoothback mutant embryos, which do not form dorsal fins as adults.

## INTRODUCTION

### Median fin evolution and development

There have been many studies on paired fins due to their undisputed homology to the paired limbs (Cope, 1890; Shubin et al., 2009; Wagner, 2014). Median fins, on the other hand, have remained relatively understudied. Extant and extinct basal vertebrates have only median fins along their bodies, and the fossil record indicates that the median caudal fin was the first appendage to appear in vertebrates, followed by the median dorsal and anal fin appendages (Shu et al., 1999; Zhang and Hou, 2004). Given that the median fins are the most ancient vertebrate appendages, it is surprising that molecular studies have only recently begun to shed light on long-standing questions about their development.

In extant fishes, median fins initiate development inside a transient midline structure called the fin fold, which forms from epidermis and periderm in early embryos (Bird and Mabee, 2003; Parichy et al., 2009). The fin fold resembles the apical ectodermal ridge of the tetrapod limb in both morphology and dependence on FGF signaling (Abe et al., 2007). The median fin fold hypothesis proposed that median fins evolved by reducing positions along the continuous fin fold of basal chordates (Larouche et al., 2019). However, a new study has demonstrated that fin fold reduction does not play a role in the establishment of the dorsal fin progenitor field (Miyamoto et al., 2022). These results align with zebrafish studies reporting aberrant fin fold mutants with predominantly normal adult fin structures (Van Eeden et al., 1996). Further, the fin progenitor field or bud, which is the mesenchymal aggregation that will give rise to the skeletal structures of the future mature fin, does not appear in the fin fold until 2 weeks post-fertilization (wpf) in zebrafish for the dorsal and anal fins (Lee et al., 2013a; Parichy et al., 2009). These mesenchymal aggregates are distinct from the population of fibroblastic mesenchymal cells that migrate from the trunk into the fin fold much earlier, at ∼30 h post fertilization (hpf), and later form the actinotrichia that provide structural support for the fin fold, and give ray-finned fish their name of Actinopterygii (Lalonde et al., 2016, 2013b). Therefore, fin folds are transient larval structures, the epithelia of which may or may not contribute substantively to adult fins, but the mesenchyme of which does not make up the skeleton of the adult fins (Lee et al., 2013b).

Throughout evolution, the dorsal and anal fins have repeatedly moved or been lost or gained in different fish clades. Some teleosts, such as sailfish, have a dorsal fin that spans almost the entirety of the antero-posterior axis of the trunk (Marras et al., 2015). Others, like walleye and perch, have two dorsal fins similar in size with a small finless gap in between (Urho, 1996). This suggests the competence for fin formation along the entire fish body axis and the existence of a repressive mechanism that prevents fins from forming ectopically. What dictates where a fin can grow along the fish body? What inhibits fin formation along the vertebrate axis? There are only a few studies addressing these questions, to our knowledge (Freitas et al., 2006; Mabee et al., 2002).

### Origin of and mechanisms establishing median fins remain mysterious

Previous studies have investigated the patterning and growth of paired appendages in fish and other vertebrates. In both median and paired fins and limbs, once the bud is established, proximodistal outgrowth is regulated by conserved FGF and BMP signaling mechanisms (Fernandez-Teran and Ros, 2008; Grandel and Schulte-Merker, 1998; Lu et al., 2008). In both the paired and median fins, Shh signaling from the posterior zone of polarizing activity (ZPA) is involved in establishing the antero-posterior axis (Harfe et al., 2004; Letelier et al., 2018; Yonei-Tamura et al., 2008; Zeller et al., 2009). Finally, genes defining the antero-posterior axis also appear to be conserved between the midline and lateral appendages, with genes like *alx4a* and *hand2* marking anterior and posterior domains, respectively, in both median and paired fins (Nachtrab et al., 2013; Yin et al., 2010). However, little is known about the earlier molecular processes that control where, when, and from what cells the median fin buds form.

The embryonic origin of the median fins has been debated for decades and was once believed to be trunk neural crest (Collazo et al., 1993; Krotoski et al., 1988; Smith et al., 1994). However, Cre lineage-tracing experiments in zebrafish demonstrated that the dorsal and anal fin rays do not contain significant neural crest contributions, but rather derive from paraxial mesoderm (somites) (Lee et al., 2013a). *tbx6* is a general marker of paraxial mesoderm (Nikaido et al., 2002). Adult transgenic *tbx6:Cre* fish carrying the *ubi:Switch* reporter present extensive lineage-labeling of the dorsal fin rays (Lee et al., 2013a,b; Mosimann et al., 2011). Some *sox10:Cre*-derived cells, of neural crest origin, were seen scattered in the caudal fin, but were not found to contribute to fin ray osteoblasts and may instead be Schwann cells. A somite transplantation study in medaka also demonstrated that paraxial mesoderm gives rise to the trunk exoskeleton, including the median fin rays (Shimada et al., 2013). Although the evidence pointing to the somitic paraxial mesoderm as the source of median fin skeleton is conclusive, the somites contain multiple compartments from which these cells could arise (Holley, 2007; Stickney et al., 2000; Tani et al., 2020). To our knowledge, no one has definitively demonstrated whether median fin skeletal cells arise from the sclerotome or dermomyotome somite compartment.

Median fin skeletal support is provided by external fin rays or lepidotrichia formed of intramembranous bone that articulate with endochondral distal and proximal radials inside the main body (Bird and Mabee, 2003). Furthermore, the skeletogenic cells that make up the fin rays of the dorsal and anal fin do not form lepidotrichia until 2-3 wpf in zebrafish (Lee et al., 2013a; Parichy et al., 2009). It is unknown where in the trunk these cells, or their progenitors, reside before dorsal and anal fin formation and after paraxial mesoderm specification at gastrulation. How the fin progenitor cells arrive at the site of the future fin and what mechanisms control the timing of this migration to the fin fold at late larval stages are also unexplored questions. Historically, these developmental processes have been harder to study due to their occurrence at later stages when the organism is no longer translucent due to squamation (Parichy et al., 2009). There is a considerable gap in knowledge and time between paraxial mesoderm specification and median fin outgrowth.

### Most fin mutants affect all fins

Fin mutants have been informative for understanding the common mechanisms for patterning and growth of all appendages. Often, mutations in appendage genes affect all fins, paired and unpaired. The *longfin* (*lof*, a regulatory allele of *kcnh2a*) (Daane et al., 2021; Iovine and Johnson, 2000; Stewart et al., 2021; Van Eeden et al., 1996) and *another longfin* (*alf*, or *kcnk5b*) mutants (Van Eeden et al., 1996; Perathoner et al., 2014) result in uninhibited growth of paired and median appendages, and all fins are reduced in size in the *short fin* (*sof*, *cx43*, or *gja1b*) mutant (Misu et al., 2016). Other mutants, such as *finless* (*fls*, or *edar*) and *nackt* (*nkt*, or *eda*), have severe phenotypes in which there are no external fin structures present along the zebrafish body because lepidotrichia fail to form, but the endochondral radials are unaffected (Harris et al., 2008). However, the mechanisms that are unique to each appendage, or class of appendage, cannot be decoupled from the general appendage program by studying mutants affecting all fins. The *eomesa* mutant is unusual in that it lacks a dorsal fin and has a reduced anal fin; the other fins are unaffected. The authors do not follow up on the striking fin phenotype (Du et al., 2012; Song et al., 2023). In carp, there are two *eomesa* co-orthologs, *eomesa1* and *eomesa2*; upon mosaic mutation of these, individuals show varying degrees of malformation in the dorsal and anal fins (Song et al., 2023). Still, no developmental mechanism or molecular insight to explain these phenotypes has been proposed for either zebrafish or carp. There also exists a medaka mutant in which a 940-bp deletion of the conserved *shh* enhancer ZRS leads to a loss of the dorsal but not the anal fin (Letelier et al., 2018). Unfortunately, no further developmental or molecular work has been reported for this mutant. In all, there is a dearth of mutant models allowing study of the early and specific mechanisms controlling median fin establishment.

Here we recovered a zebrafish dominant transgenic insertional mutant in which the dorsal fin is absent. Mechanistically, this phenotype is caused by insertion of a transgene containing a *sox10* promoter driving *Gal4VP16* (hereafter *sox10:Gal4*) into a non-coding region. We have termed this mutant *smoothback* (*smb*), due to the absence of dorsal fin skeletal structures. While the dorsal fin is fully absent in the majority of individuals, with some variation in expressivity, the anal fin is disorganized and posteriorly truncated. All other fins are overtly unaffected in *smb* heterozygous mutants. To investigate the cause of the missing dorsal fin bud mesenchyme, we revisited the question of which somite compartment gives rise to median fin progenitors. We conclusively show for the first time that sclerotome cells are the source of the median fin skeletal cells. Consistently, we find that there is reduced sclerotome signal in *smb* heterozygous mutants compared to wild-type embryos, and propose a model of early sclerotome depletion in *smb* heterozygous mutants that explains the missing and truncated median fins. Our results give insight into the understudied developmental mechanisms that establish the dorsal and anal fins.

## RESULTS

### *smb* is a dominant mutant with median fin phenotypes caused by a transgenic insertion

**Fig. 1.**
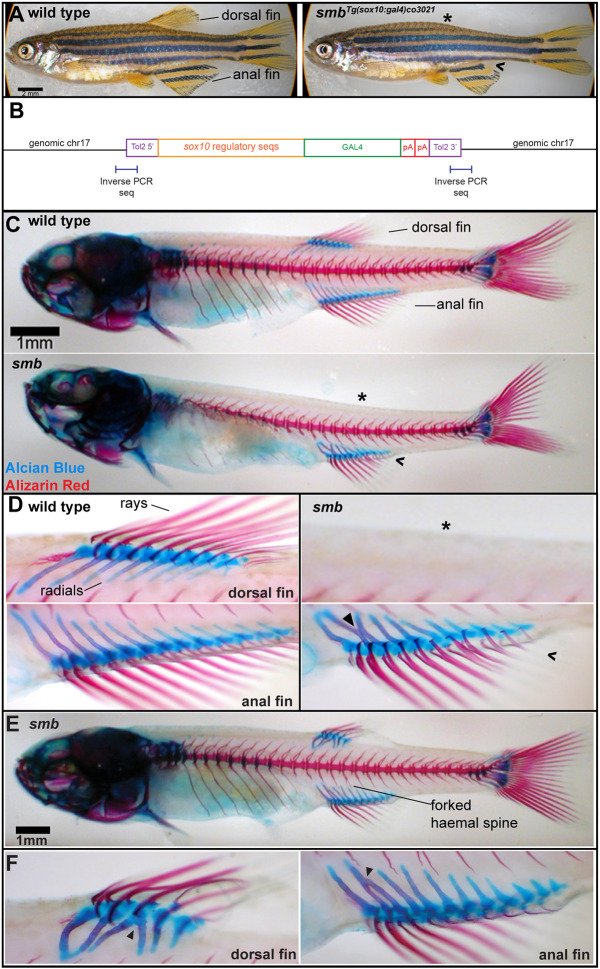
**The insertional *smb* mutant presents median fin phenotypes.** (A) Whole mount wild-type adult (*n*=50) and heterozygous *smb^Tg(sox10:Gal4)co3021^* (*n*=50) zebrafish (hereafter *smb* mutants) were live imaged with transmitted light. In all panels, asterisk denotes the missing dorsal fin; caret indicates reduced size anal fin. (B) Schematic indicating the transgenic sequence and genomic location of the *sox10:Gal4* insertion in the *smb* background. (C) Wild-type and *smb* heterozygous mutant juveniles (33 dpf) were stained with Alcian Blue (cartilage) and Alizarin Red (bone) and imaged by light microscopy. (D) Enlargements from panel C depicting the radials and rays of the dorsal and anal fins in wild types and mutants. Arrowhead indicates disorganized anal fin radials in mutants. (E) Representative *smb* heterozygous mutant with partial fin loss was imaged with transmitted light. (F) Arrowheads in enlargements indicate fused radials in reduced dorsal and anal fins. Scale bars: 2 mm (A); 1 mm (C,E).

We fortuitously recovered the *smb^Tg(sox10:Gal4)co3021^* transgenic mutant allele while creating a *sox10:Gal4* transgenic line using the Tol2 system (Kwan et al., 2007). Hereafter, we refer to this allele as the *smb* mutant. We first identified potential transgenic founders (G0) after injection of the *sox10:Gal4* construct into *UAS:E2Crimson* transgenics by mosaic expression in neural crest cells. Several different potential founders also carrying *UAS:E2Crimson* were raised to adulthood and outcrossed to AB wild types generating different F1 families, likely each with a different integration of the transgene. *sox10:Gal4* F1 carriers from different G0 founders were identified by consistent E2Crimson expression and raised to adulthood and subsequently crossed to wild types generating different F2 families. We observed that one of these transgene-carrying F2 families lacked dorsal fins and had reduced anal fins along the antero-posterior (AP) axis in all transgenic (*sox10:Gal4*^+^) adults (Fig. 1A). The loss of dorsal fin and anal fin structures is 100% penetrant, though there is variation in expressivity of dorsal fin loss. The loss of dorsal and anal fin structures phenotype never appeared in non-transgenic siblings, nor in any families from other founders injected with the same construct. All other fins, caudal and paired, had no overt phenotype in mutants. In heterozygous *smb* to wild-type crosses, 50% of the offspring presented median fin phenotypes, suggesting that this mutant is inherited as a Mendelian dominant allele (*n*=100). For many generations, we maintained the *smb* line by outcrossing to wild type and selecting phenotypically *smoothback* animals to propagate the next generation. After ten generations of this selection strategy, we crossed *smb* heterozygous mutants to *UAS:E2Crimson* fish to determine if the *smb* mutants were still carriers of the *sox10:Gal4* transgene. Indeed, the transgene was still present in the *smb* background, and in this cross all *sox10:Gal4* transgenic animals developed the median fin phenotype, while all non-*sox10:Gal4*-transgenic siblings developed wild-type fins. That the transgene was still present in *smb* fish after many rounds of outcrossing and selecting for just the mutant phenotype suggests that the transgene is either causative of the loss of fin phenotype or tightly genetically linked to the causative genomic feature.

### Homozygous *smb* mutants have further reduced anal fins

To determine whether *smb* mutant homozygosity exacerbates the heterozygous *smb* mutant fin phenotypes, we intercrossed heterozygous fish. At adult stages, we observed that only 25% of their offspring had full dorsal fins. The other 75% presented dorsal fin loss and reduced anal fins. However, we noticed that some individuals from this family had anal fins that appeared further reduced in size along the AP axis compared with others in the clutch (Fig. S1). Upon genotyping by PCR, we confirmed that the fish with the shortest anal fins were homozygous *smb*. We quantified this defect by counting anal-fin rays. Whereas all wild-type fish had 15 anal fin rays, heterozygous *smb* fish varied between ten and 11, and *smb* homozygous mutants consistently had only eight (Fig. S1). This finding indicates a more severe anal fin ray phenotype with the addition of a second *smb* mutant allele.

### The *sox10:Gal4* transgene in *smb* mutants drives Gal4 in diverse tissues and does not disrupt an annotated coding sequence

The *smb* transgene contains *sox10* regulatory sequences (∼3.1 kb upstream of the annotated start codon). *sox10* is a marker of neural crest cells (NCCs), and *sox10* regulatory sequences are well characterized to drive transgenic expression in NCCs and their derivatives, such as the cartilage of the face, neurons, and glia (Kelsh, 2006; Takada et al., 2010). Other reported *sox10* regulatory sequences drive expression of *Gal4* in these NCC derivatives. To test if our *sox10:Gal4* transgene insertion in *smb* mutants is faithful to NCC expression, we crossed an *smb* heterozygous mutant to *UAS:E2Crimson* fish to visualize *Gal4* activity and imaged the double transgenic *smb*(*sox10:Gal4*)*;UAS:E2Crimson* fish at 28 hpf. We noticed labeling of pharyngeal arch NCCs and the neural tube by our transgene (Fig. S2A), as expected (Cunningham et al., 2021). Interestingly, we also observed expression in the mesoderm of the trunk and tail bud (Fig. S2A). Other *sox10:Gal4* constructs do not label cells in these tissues (Lee et al., 2013b). Expectedly, we found that our transgene labels NCC derivatives, such as the Meckel's cartilage, hyosymplectic, and ceratohyal craniofacial cartilage elements at 5 days post-fertilization (dpf), as evident by co-expression of *E2Crismson* and *sox9a:EGFP*, a bona fide cartilage marker (Eames et al., 2013) (Fig. S2B).

To determine if the mesoderm expression persists later in development, we also examined the fin folds of *smb* fish at 5 dpf. We found that the mesoderm-derived fibroblasts (Lee et al., 2013b) that populate the larval fin folds are labeled by our transgene in the *smb* strain (Fig. S2C); however, other published *sox10:Gal4* lines do not express *Gal4* in these cells (Lee et al., 2013a,b). It is important to note that the *sox10* regulatory sequences in our construct are shorter (∼3.1 kb) than the previously reported ones (7.2 and 4.9 kb) (Kucenas et al., 2008; Lee et al., 2013b). At later stages (5.1 mm standard length, SL), we also observed Gal4 activity in the anal fin bud mesenchyme of *smb* heterozygous mutants (Fig. S2D). No Gal4 activity was detected in the region of the fin fold where the dorsal fin bud should form, suggesting that the mesenchyme itself may be absent.

Transgenes such as *Gal4* can be toxic and detrimental to tissues (Gill and Ptashne, 1988; Kimmel et al., 2021). Thus, we hypothesized that *Gal4* expression is deadly to dorsal fin progenitor cells. To address this, we injected the same *sox10:Gal4* transgenic construct causative of the *smb* phenotype into the *UAS:E2Crimson* line. We generated three new stable *sox10:Gal4* transgenic lines with likely independent integrations. We found that wild-type dorsal and anal fins developed in all new stable integrants, even those that expressed Gal4 in the dorsal and anal fins (Fig. S2E). The consistent fin expression across multiple insertions of the same transgene likely differs from endogenous *sox10* and other *sox10* transgenic construct expression patterns because the regulatory sequences in our construct are shorter than other constructs. We concluded that the *smb* phenotype is genomic integration site-dependent and is not strictly due to Gal4 protein expression in fin mesenchyme. These results motivate the hypothesis that *sox10:Gal4* transgene integration specifically into the *smb* genomic locus affects endogenous gene expression, or gene function, or both to cause median fin phenotypes in *smb* mutants.

To determine where the *smb* causative transgene integrated, we used an established mapping method (Eames et al., 2013). We performed inverse PCR using the restriction sites found in *tol2* arm sequences from the transgene as previously described. We localized both the 5′ and 3′ ends of the transgene to chr17:46,219,972 of the zebrafish genome (GRCz11/danRer11) (Fig. 1B). This is an intergenic region 200 kb away from the nearest annotated gene, *si:dkey-206p8.1*, an uncharacterized open reading frame. Insertion into a gene desert means that the *sox10:Gal4* transgene insertion in *smb* mutants is not interrupting an annotated gene or ncRNA and therefore likely has a more complex molecular mechanism of action, currently under investigation.

### The dorsal fin skeleton fails to develop and anal fin skeletal elements are disorganized in smoothback mutants

We wished to further characterize the *smb* mutant phenotype. To determine if only the externally visible fin rays are absent in *smoothback*, as in *fls* mutants, or if the internal radials are also affected, we stained heterozygous *smb* mutants and wild-type siblings with Alcian Blue and Alizarin Red to label cartilage and bone, respectively. We found that both the external fin rays and internal endochondral radials that comprise the dorsal fin in wild-type controls were absent in heterozygous *smb* mutant adults (Fig. 1C,D). The dorsal fin loss is partial in some families (Fig. 1E,F), but this milder phenotype is rare (15% of *smb* heterozygous mutant fish) and expressivity varies among families (Tables 1 and 2). We therefore focused on the severe phenotype, the complete absence of the dorsal fin. In the anal fin, mutants showed a reduced number of rays and radials with 100% penetrance. We also noticed frequent disorganization and fusions of anal cartilage radial elements (Fig. 1D,F; Fig. S1).

**Table 1.**
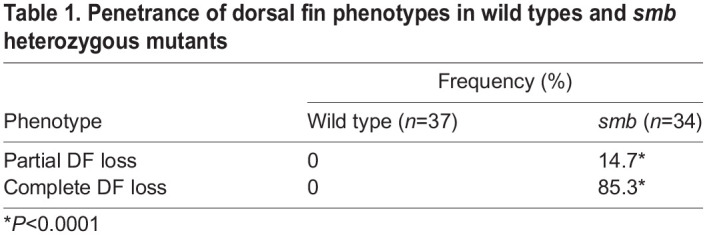
Penetrance of dorsal fin phenotypes in wild types and *smb* heterozygous mutants

**Table 2.**
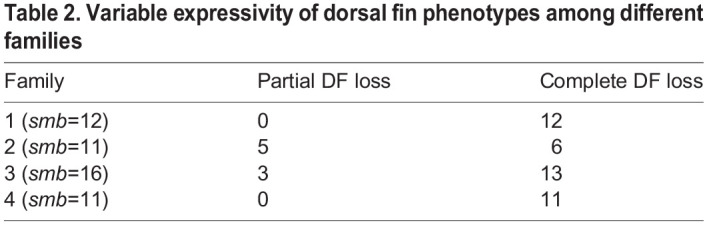
Variable expressivity of dorsal fin phenotypes among different families

**Fig. 2.**
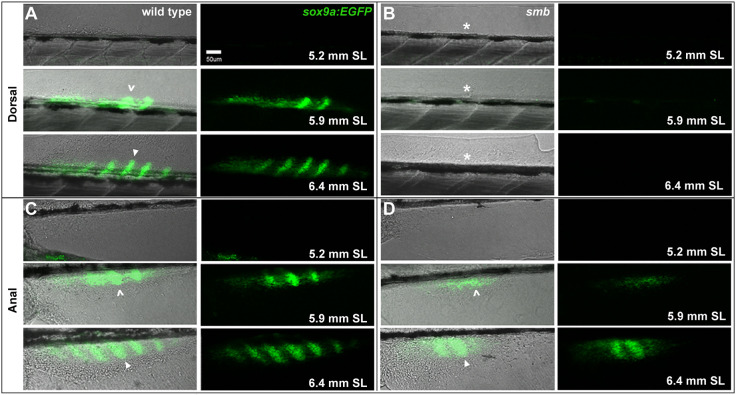
**The chondrogenesis program does not initiate in *smb* dorsal fins.** (A-D) Heterozygous *smb* mutants were crossed to *sox9a:EGFP* fish, and offspring were imaged by confocal and transmitted light microscopy during progressive stages of median fin chondrogenesis. (A) In wild-type dorsal fins, the caret indicates initiation of the chondrogenesis program marked by EGFP; white arrowhead indicates stacks of chondrocytes in the developing radials. (B) In *smb* heterozygous mutants, the asterisk indicates absence of dorsal fin bud mesenchyme and the chondrogenesis program. (C) In wild-type anal fins, the caret indicates initiation of the chondrogenesis program marked by EGFP; white arrowhead indicates stacks of chondrocytes in the developing radials. (D) In *smb* heterozygous mutant anal fins, the caret indicates initiation of the chondrogenesis program marked by EGFP. White arrowhead indicates stacks of chondrocytes in the developing radials. Fewer individual radials form in mutants. Scale bar: 50 μm.

To examine earlier stages of skeletal formation in the dorsal and anal fins, we crossed *smb* heterozygous mutants to *sox9a:EGFP* to assess the developing median-fin cartilage radials (Eames et al., 2013), and performed a developmental series comparing wild type and *smb* heterozygous mutants (Fig. 2). At 5.2 mm SL, there was no signal in the dorsal fin folds of wild-type or *smb* heterozygous mutant fish. By 5.9 mm SL, developing dorsal fin radials were evident in wild types, but there were no comparable EGFP^+^ structures in mutants. This same pattern is seen at 6.4 mm SL; dorsal *sox9a:EGFP*-expressing radials were well-developed in wild types but missing in mutants (Fig. 2A,B). Similarly, in the anal fin region of the ventral fin fold, there was no chondrocyte signal in either wild-type or *smb* heterozygous mutant fish at 5.2 mm SL. At 5.9 mm SL, wild-type fish had developing anal fin radials. While there are EGFP^+^ structures in mutant post anal fin folds, they appeared to be reduced in number. Finally, at 6.4 mm SL, we observed well-developed *sox9a:EGFP*-expressing radials in anal fins of wild types but fewer in *smb* heterozygous mutant fish (Fig. 2C,D).

We next stained the musculature of 6.8 mm SL fish using Phalloidin. Whereas the rays of wild-type median fins are connected individually through musculature to the trunk, we observed disorganization of muscle fibers in the anal fin of *smb* heterozygous mutants (Fig. S3). For example, this mutant presents an ectopic muscle fiber connecting the erectores anales of ray 1 to the depressores anales of ray 5. We did not observe any musculature in the absent dorsal fin of *smb* heterozygous mutants. Together, these results indicate that *smb* heterozygous mutant fish have musculoskeletal phenotypes limited to the median fins, specifically the dorsal and anal fin, but no other gross developmental defects.

### Fin development blockade in *smb* occurs upstream of fin outgrowth

**Fig. 3.**
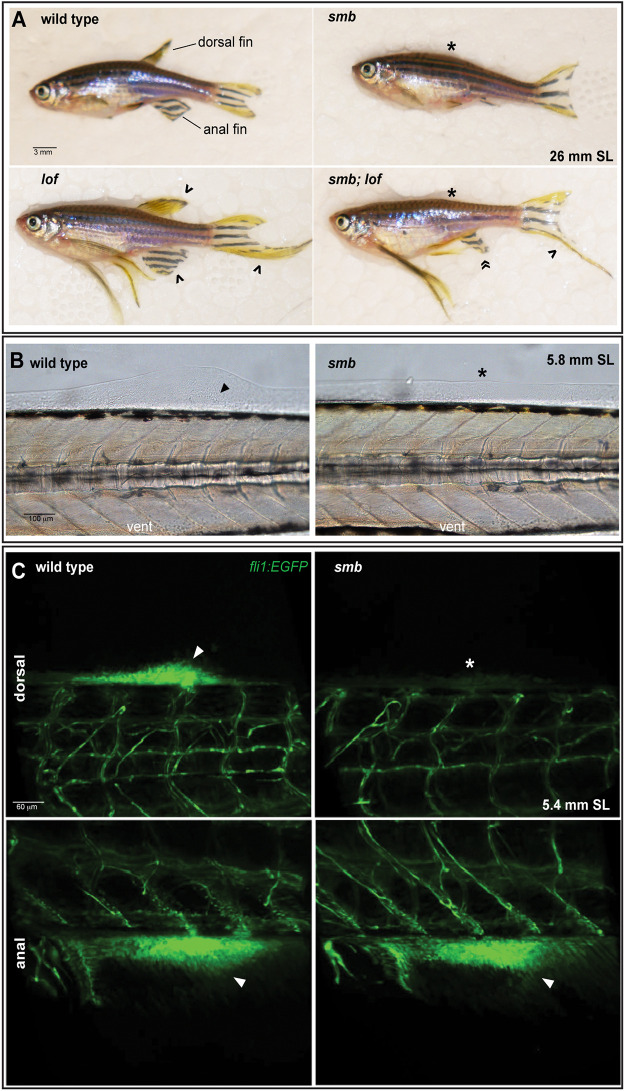
**Establishment of dorsal fin bud progenitors is necessary for fin outgrowth.** (A) The *smb* mutant is epistatic to *lof*. Heterozygous *smb* adults were crossed to heterozygous *lof* adults and adult offspring were imaged with transmitted light. The dorsal fin and anal fin are indicated in the wild-type (wt) animal. An asterisk indicates an absent dorsal fin, a caret indicates elongated fins, and guillemets indicate an anal fin that is reduced in the AP axis but elongated in the proximodistal axis in *smb*;*lof* double heterozygous mutants (wt=8, *smb*=4, *lof*=8, *smb;lof*=5, expected=6.5 per genotype. Chi-square test; *P*=0.5641). (B) Nomarski images of fin induction and mesenchyme aggregation in wild type and *smb* heterozygotes at 5.8 mm SL (*n*=3 per genotype). Arrowhead indicates fin bud mesenchyme, asterisk marks absent fin bud. (C) Confocal images of live 5.4 mm SL *fli1:EGFP* wild-type and *smb* heterozygous mutants (*n*=10 per genotype). Arrowhead indicates EGFP-expressing mesenchyme cells in the dorsal and anal fin buds. Asterisk indicates absence of *fli1:EGFP* cells in the dorsal fin bud in *smb* heterozygous mutants. Scale bars: 3 mm (A); 100 μm (B); 60 μm (C).

Epistasis, in which a double mutant phenotype is compared with those of single mutants, can inform gene hierarchy even without knowledge of the nature of the mutations, the pathway, or molecular mechanisms (Avery and Wasserman, 1992). Fish carrying the *lof* allele have increased allometric growth of all fin appendages (Daane et al., 2021; Iovine and Johnson, 2000; Stewart et al., 2021; Van Eeden et al., 1996). We reasoned that the *lof* mutant might genetically interact with the *smb* mutant. We performed an epistasis analysis by crossing a heterozygous *smb* mutant to a *lof* heterozygous mutant (Fig. 3A) and growing the offspring to adulthood (23 mm SL) to compare fin phenotypes between single and double mutants. We saw four different phenotypes in expected Mendelian ratios in the adults. The wild types had all median and paired fins at the expected fin size. The *smb* heterozygous mutants did not have a dorsal fin and the anal fin was reduced, but fin growth was isometric. All fins in heterozygous *lof* single mutants were elongated, as previously reported (Stewart et al., 2021). Fish heterozygous for both *smb* and *lof* had elongated paired and caudal fins but no dorsal fins. Strikingly, the anal fin of these fish was elongated in the proximo-distal axis but remained reduced along the AP axis (Fig. 3A, guillemets). Thus, our *smb* mutant is epistatic to the *lof* mutant; no fin skeletal structures will grow where the fin is missing, but the other appendages will have prolonged fin growth. These results suggest that the *smb* heterozygous insertion disrupts fin development upstream of fin elongation and is perhaps involved in the earliest stages of fin formation.

### *smb* mutants do not establish the dorsal fin bud

Our epistasis experiment indicates that the developmental defect in *smb* heterozygous mutants occurs early in fin development. Moreover, *smb* heterozygous mutants develop defects in both the endochondral radials and the dermal fin rays. These results motivate the hypothesis that the *smb* insertion potentially disrupts one of the earliest reported steps in median fin formation, establishment of the fin bud mesenchymal aggregation, or fin induction. The earliest stage of dorsal fin induction is a small outgrowth in the dorsal larval median fin fold (LMFF) at ∼5.6 mm SL (Miyamoto et al., 2022). Using Nomarski microscopy, we found that this LMFF outgrowth is not present in *smb* heterozygous mutants compared to wild-type siblings. Nomarski microscopy further revealed that no mesenchymal aggregation appears in mutants compared to wild type (Fig. 3B). To get a better view of the dorsal fin bud mesenchyme aggregation we next used confocal microscopy. The *fli1:EGFP* transgene labels the mesenchymal progenitors of the facial skeleton as well as the vasculature (Lawson and Weinstein, 2002). We hypothesized that *fli1:EGFP* might also label the undifferentiated mesenchymal progenitors of the fin skeleton. Indeed, we found that *fli1:EGFP* is strongly expressed in the dorsal and anal fin mesenchymal progenitor fields in wild-type fish at 5.4 mm SL (Fig. 3C). In contrast, stage-matched *smb* heterozygous mutants had no *fli1:EGFP*-expressing dorsal mesenchyme but did form anal mesenchymal aggregates. These results support our hypothesis that, in *smb* heterozygous mutants, the earliest reported step in median fin formation, induction of fin bud mesenchymal progenitors at the site of the future dorsal fin, is impaired.

### Posterior anal fin mesenchyme is missing in *smb* mutants

**Fig. 4.**
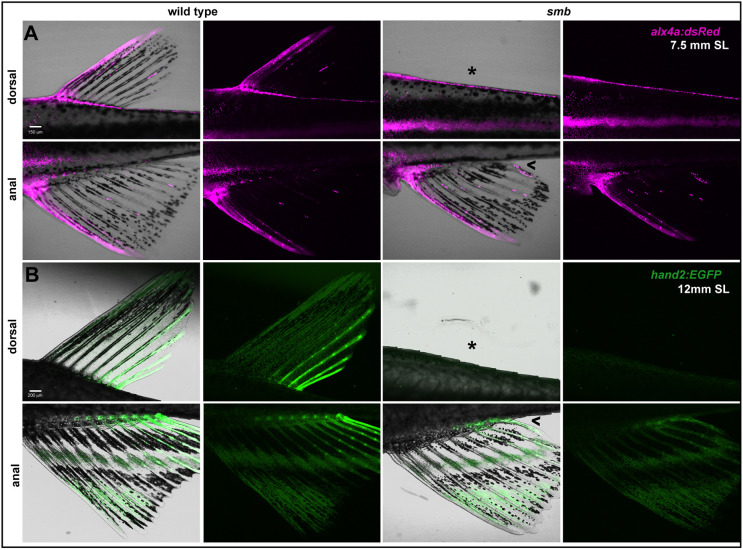
**The posterior domain of the anal fin is sensitive to the *smb* insertion.** (A) Anterior median fin rays labeled with *alx4a:dsRed* were live imaged in wild-type and *smb* heterozygous mutants (*n*=3 per genotype)*.* (B) Posterior median fin rays labeled with *hand2:EGFP* were live imaged in wild-type and *smb* heterozygous mutants (*n*=3 per genotype). In both panels, asterisk denotes absent dorsal fin, caret indicates reduced anal fin. Scale bars: 150 μm (A); 200 μm (B).

In contrast to the dorsal fin, the anal fin of *smb* heterozygous mutants was not completely absent, but rather reduced along the AP axis. Because the mesenchyme of the anal fin appeared to be reduced at the posterior end in our heterozygous *smb* fish carrying *fli1:EGFP* (Fig. 3C), we wished to determine whether this same population was missing from the adult anal fin of *smb* heterozygous mutants. We crossed heterozygous mutants to transgenic lines labeling the anterior (*alx4a:DsRed*) and posterior (*hand2:EGFP*) rays of median fins (Kikuchi et al., 2011; Nachtrab et al., 2013). The first two anterior-most rays of the dorsal and anal fins were labeled by *alx4a:DsRed* in wild-type zebrafish (Fig. 4A). In *smb* heterozygous mutants, we saw no expression of *alx4a:DsRed* dorsally given that there was no dorsal fin. The first two anterior-most rays of the anal fin in *smb* heterozygous mutants were also labeled by this transgene just like in the wild types. In wild types, we observed that *hand2:EGFP* labeled the posterior-most 4-5 rays of the dorsal fin and the last 8-9 rays of the anal fin (Fig. 4B). In severe *smb* heterozygous mutants, there was no dorsal expression of *hand2:EGFP* as there was no dorsal fin. In the anal fin of *smb* heterozygous mutants, there was a significant decrease in the number of posterior rays (*P*=0.0006); only the last 4-5 rays were *hand2:EGFP*^+^. Similarly, in the partial dorsal fins of mild *smb* heterozygous mutants, we observed a decrease in the number of *hand2:EGFP*^+^ radials compared to wild-type siblings; the average number of posterior rays in wild types was 5, whereas this number decreased to 1.5 in these mild heterozygous mutants (Fig. S4). This finding supports the hypothesis that the posterior-most domain of the dorsal and anal fins – which form last (Mabee et al., 2002) – are preferentially lost in *smb*.

### Early AP patterning appears to be unaffected in *smb* mutant embryos

To better understand the preferential loss of posterior structures in the dorsal and anal fins of *smb* mutants, we probed for early axial AP patterning defects in mutant embryos. Axial expression patterns of hoxd genes show the expected collinear expression in the mesoderm of zebrafish embryos at ∼22 hpf (van der Hoeven et al., 1996) and hox gene function was recently demonstrated to affect position and size of dorsal and anal fins (Adachi et al., 2024). *hoxd12* shows an expression domain with an anterior border at somite 17, which marks the region of the anterior margin of the anal fin and the posterior half of the dorsal fin. We performed Hybridization Chain Reaction (HCR) for RNA localization (Choi et al., 2014), probing for *hoxd12* in 23 hpf wild-type and *smb* heterozygous mutant embryos. We found the expected *hoxd12* expression pattern starting posterior to the vent in wild-type embryos as well as *smb* heterozygous mutants; the expression pattern of this hox gene was unchanged in the mutants (Fig. S5). These results indicate that the dorsal and anal fin phenotypes in *smb* are primarily due to a fin induction defect and not secondary to irregular anteroposterior patterning, which appears to be unaffected in *smb* heterozygous mutants.

### The sclerotome is the source of the median fin skeleton

**Fig. 5.**
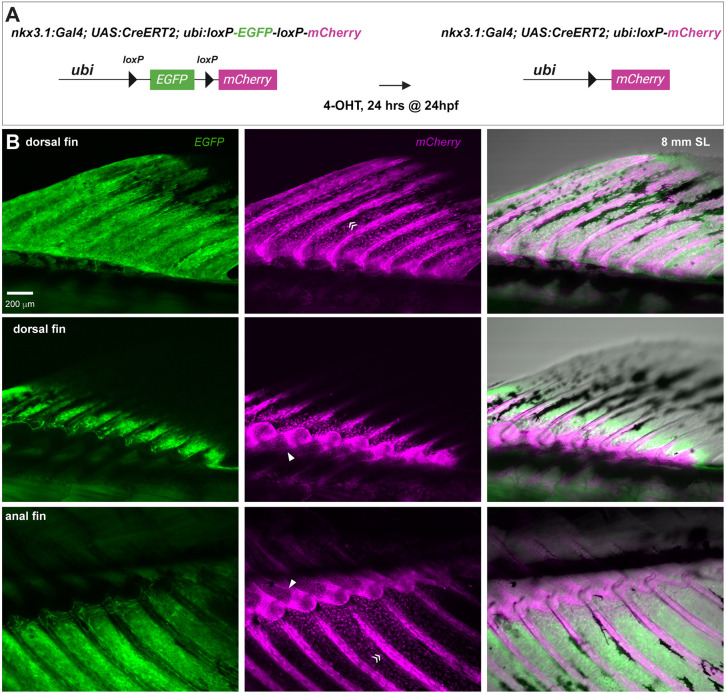
**The sclerotome is the source of the dorsal and anal fin skeleton.** (A) Schematic indicating transgenic lines and tamoxifen treatment protocol used to lineage trace the sclerotome using *nkx3.1:Gal4*. (B) Sclerotome-derived, Cre-recombined, mCherry-expressing cells in the dorsal and anal fins were imaged. Arrowheads indicate median fin radial chondrocytes; guillemets indicate median fin ray osteoblasts. *n*=12/12 individuals with recombination in sclerotome labeled the dorsal and anal fins. Scale bar: 200 μm (B).

To uncover the earliest manifestation of the *smb* phenotype, we decided to further our understanding of median fin development. Although the lineage of the median fins has been traced back to the paraxial mesoderm (Lee et al., 2013a; Shimada et al., 2013), there is still no conclusive evidence about which somite compartment is the source of fin progenitors. We chose to look at the sclerotome first as it gives rise to the bones of the ribs and vertebral spines and thus has skeletogenic potential (Ma et al., 2023; Tani et al., 2020). To achieve this, we crossed *nkx3.1:Gal4;UAS:CreERT2* to *ubi:Switch* to specifically lineage trace sclerotome derivatives (Ma et al., 2018). *nkx3.1* is a bona fide marker of the sclerotome and at 24 hpf robustly labels both the dorsal and ventral domains of the bipartite zebrafish sclerotome. We treated triple transgenic fish at 24 hpf with 4-hydroxytamoxifen (4-OHT) for 24 h, driving *EGFP* to permanently switch to *mCherry* in *nkx3.1*-expressing cells during this narrow window of development (Fig. 5A). At 4 dpf, we observed successful labeling of sclerotome-derived cells, including tenocytes, fin mesenchymal cells, and other fibroblasts, consistent with previous lineage analyses (Ma et al., 2018, 2023). At 8 mm SL, during late median fin development, we observed *mCherry*^+^ osteoblasts and chondrocytes within the rays and radials of the dorsal and anal fins (Fig. 5B; Fig. S6). Our findings conclusively show that the sclerotome is the source of the mesenchyme that gives rise to the rays and radials of the dorsal and anal fin. Unfortunately, because the *smb* line contains its own *Gal4* construct, we could not reproduce the sclerotome tracing experiment in the mutant.

### Sclerotome expansion is decreased in *smb* mutants

**Fig. 6.**
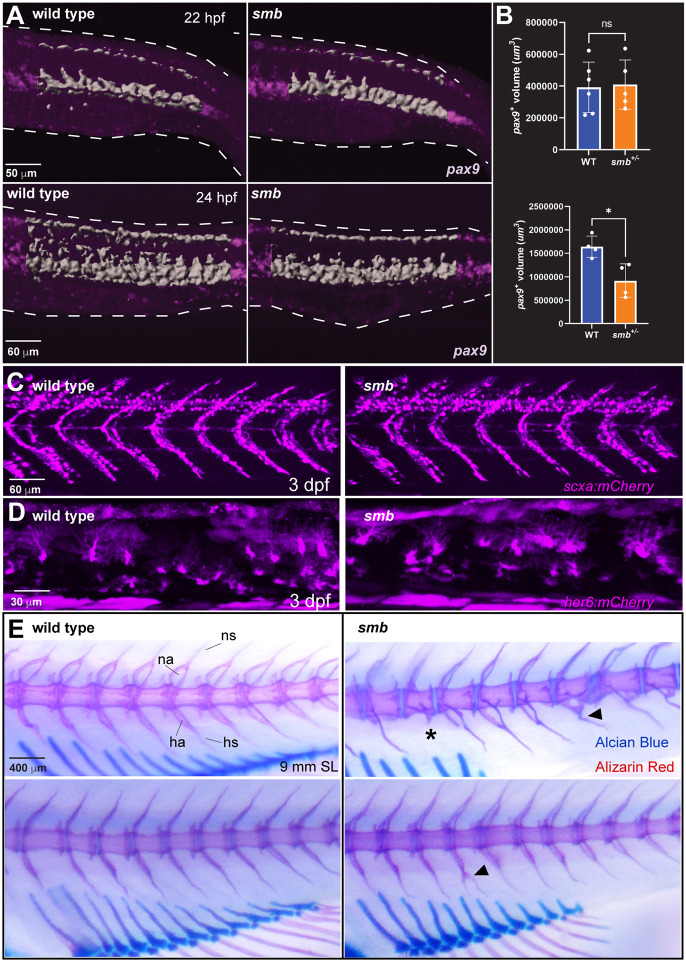
***smb* mutants have impaired sclerotome expansion but early-forming derivatives of the sclerotome are unaffected in the *smb* mutant.** (A) *pax9* transcripts were fluorescently labeled with HCR in wild-type and *smb* heterozygous embryos at 22 and 24 hpf and imaged by confocal microscopy. Gray indicates the *pax9*^+^ domain used to quantify volume using Imaris surface rendering. (B) *pax9* expression at each condition was quantified by Imaris. For 22 hpf, six wild-type and six *smb* heterozygous mutant animals were quantified (unpaired two-tailed *t*-test; *P*=0.8640; ns, not significant). For 24 hpf, four wild type and four *smb* heterozygous mutants were quantified. *P=0.0144; unpaired two-tailed *t*-test. (C) Heterozygous *smb* fish were crossed to *scxa:mCherry* fish, and labeled tenocytes in wild-type and *smb* heterozygous mutant offspring were imaged by confocal microscopy (*n*=5 per genotype). (D) *smb* heterozygous fish were crossed to *her6:mCherry* fish, and fin fold fibroblasts in wild-type and *smb* heterozygous mutant offspring were imaged by confocal microscopy (*n*=3 per genotype). (E) Vertebrae were imaged in Alcian Blue and Alizarin Red-stained wild types and stage-matched *smb* heterozygous mutants. Neural spines (ns), neural arches (na), haemal spines (hs), and haemal arches (ha) are indicated in wild type. Asterisk indicates absent haemal arch and spine; arrowheads mark fused or forked haemal arches or spines. Fisher's exact test; *P*<0.0001. Scale bars: 50 μm (A, top); 60 μm (A, bottom, C); 30 μm (D); 400 μm (E).

After tracing the origin of the dorsal and anal fins to the sclerotome, we decided to investigate the establishment and development of this source tissue in our mutants to assess any early developmental phenotypes. Ma et al. (2018) showed that *pax9* and *nkx3.1* are markers of the bipartite zebrafish sclerotome. Using *pax9* as a sclerotome marker, we performed HCR (Choi et al., 2014) to investigate *pax9* expression domains in wild type and *smb*. To assess differences, we quantified the *pax9*^+^ volume of the five somites immediately posterior and anterior to the vent, the same AP axis that is later spanned by the dorsal and anal fins. This analyzed region includes the dorsal and ventral domains of the sclerotome, including the sclerotome-derived notochord-associated cells from the ventral domain. We found that at 22 hpf there were no differences in *pax9* expression between wild type and *smb* heterozygous mutants (Fig. 6A,B). Surprisingly, by 24 hpf there was a significantly lower volume of *pax9* expression in *smb* heterozygous mutants compared to wild-type siblings (Fig. 6A,B). These differences were observed in both the dorsal and ventral domains when analyzed separately (Fig. S7). When we examined the sclerotome at a slightly later stage, we observed a decrease in only the dorsal, but not the ventral, domain of the sclerotome in *smb* heterozygous fish compared to wild types at 26 hpf (Fig. S8). By 30 hpf, there were no differences in *pax9* expression between wild type and *smb* heterozygous mutants (Fig. S9). However, the sclerotome is a transient embryonic tissue and is no longer bipartite at this stage. Furthermore, sclerotome-derived cells have begun to migrate dorsally into the somite boundaries by 30 hpf. Thus, *pax9* no longer specifically marks the bipartite sclerotome at this stage. Nevertheless, our results suggest that although the sclerotome is properly established, there is a defect in the expansion of this tissue per *pax9* expression in *smb* heterozygous mutants and that this defect persists later in the dorsal domain of the sclerotome.

### Early sclerotome derivatives remain unaffected in the *smb* mutant

We analyzed other known sclerotome derivatives for changes in the *smb* mutant. By 2 dpf, tenocytes are present along myotendinous junctions (MTJs) at somite boundaries (Ma et al., 2018). We used *scxa:mCherry* (McGurk et al., 2017), a tenocyte reporter, to look for changes in sclerotome-derived tenocytes. We found no differences in the number or intensity of these structures between stage-matched wild types and *smb* heterozygous mutants at 3 dpf (Fig. 6C). Next, we looked at the fin fibroblasts of the fin fold at 3 dpf, which we find are labeled by the *her6:mCherry* transgene (Kraus et al., 2022). We observed no overt differences in number or shape of these cells between wild types and *smb* heterozygous mutants (Fig. 6D)*.* Finally, we looked at the vertebral bodies of the zebrafish spine using Alizarin Red as a label. Surprisingly, we found a significant increase in haemal spine abnormalities in *smb* heterozygous fish compared to wild types. The phenotypes quantified for this analysis include any forking of the haemal spine, missing spines, and spine fusions at the axial level of the anal fin (Fig. 6E; Table 3). Taken together, these results show that derivatives of the sclerotome that emerge at early larval stages are unaffected in the *smb* mutant, while the later forming structures like the vertebral spines and median fins are more sensitive to the *smb* mutation.

**Table 3.**
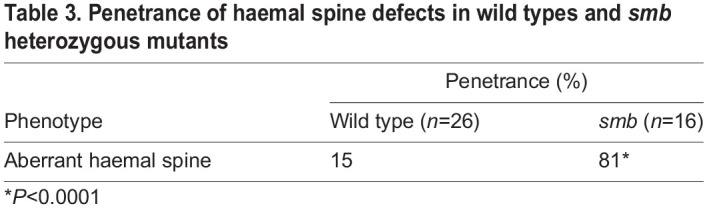
Penetrance of haemal spine defects in wild types and *smb* heterozygous mutants

## DISCUSSION

Much emphasis has been placed on the fin-to-limb transition during vertebrate evolution (Hawkins et al., 2021; Shubin et al., 2008; Wagner, 2014). By contrast, the permanent loss of dorsal and anal fins during the sea-to-land transition was a concurrent major shift in the vertebrate body plan that has remained poorly studied and largely overlooked by the research community. No longer faced with the constant challenge of maintaining body posture against shifting water currents, early tetrapods like *Tiktaalik* lost their stabilizing midline fins as they evolved more limb-like paired appendages (Shubin et al., 2006). In the case of whales and dolphins, there was subsequently convergent evolution of a dorsal ‘fin’ structure when these mammals returned to an obligate aquatic lifestyle; however, this fin lacks the endoskeletal support found in fish (Gatesy et al., 2013). It is likely that these mammalian dorsal fins arose through the evolution of different developmental mechanisms.

In addition to this permanent loss in the tetrapod lineage, median fins have also frequently changed positions and been gained and lost along the body axis among fish species. Even within fish species, some natural losses of the dorsal fin have been documented. For example, a cutthroat trout population lacking dorsal fins was found in Utah in 1937 (Code, 1950). Others arise in the pet-trade, like the eggfish, a goldfish with no dorsal fin (Andrews, 2002). Dorsal fins are seemingly not required for viability under laboratory settings but are likely under selective pressure due to utility in courtship and reproduction. In one zebrafish study, researchers found that the dorsal fin is required in female zebrafish for males to hook their body and compress the female for eggs. This behavior is known as ‘hooking’. By physically removing the dorsal fin in females, the authors found that males could no longer hook and would slip from the female's trunk (Zempo et al., 2021). Accordingly, we have found that it is difficult to get *smb* heterozygotes to breed with each other. For this reason, we largely focused on the slightly less severe dominant phenotypes seen in *smb* heterozygotes, as an *smb* male will readily breed with a wild-type female.

In the context of our mutant, timing of fin development likely underlies the specificity of the phenotype. The caudal fin forms first at 3 dpf and is unaffected by the *smb* insertion. The anal fin forms second at 5.4 mm SL (Parichy et al., 2009) and is only partially affected in *smb* heterozygous mutants. Within the anal fin, the early-forming anterior end appears to be less affected compared to the more sensitive and later-forming posterior segment, in which both radials and rays are gone. A comparative study revealed the recurrence of similar developmental patterning in the dorsal and anal fins. This refers to the sequences and direction of development among endoskeletal and exoskeletal elements in the dorsal and anal fins among living actinopterygians (Mabee et al., 2002). These authors interpreted the similar patterning of the dorsal and anal fins as indicative of an Endoskeleton and Exoskeleton Module, where the directions of development (AP axis) of the endoskeleton (radials) and exoskeleton (lepidotrichia) are similar. The posteriorly-truncated anal fin and posteriorly-truncated partial dorsal fins in *smb* mutants are fitting of the Endoskeleton and Exoskeleton Module proposed by Mabee et al. (2002). In the more severe phenotypes, the latest-forming (5.7 mm SL) dorsal fin (Parichy et al., 2009) is the most sensitive to the *smb* insertion.

**Fig. 7.**
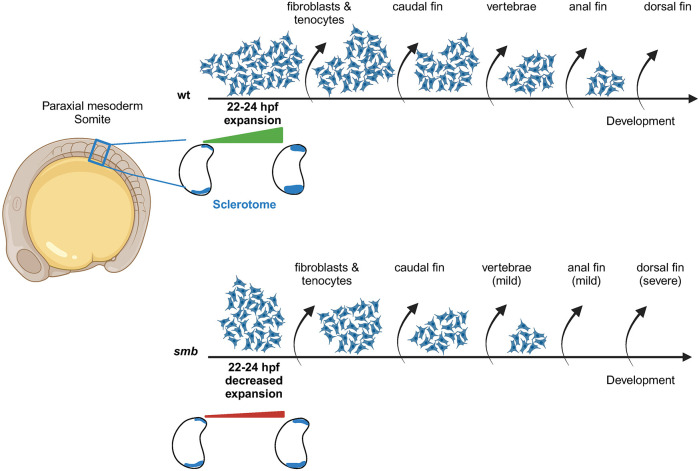
**Failed sclerotome expansion and early depletion may underlie fin loss in *smb* mutants.** The sclerotome is specified in the somites at ∼20 hpf, and in wild types expands from 22-24 hpf. Later, this compartment gives rise to multiple tissues in the fish trunk. We propose that as development progresses and tissues are formed, this progenitor pool becomes depleted. In *smb*, impaired expansion of the sclerotome leads to a smaller initial pool of progenitors, which depletes earlier, leaving no cells to build late-forming structures like the posterior region of the anal fin and the dorsal fin.

The two affected fin buds represent the final major structures formed from the sclerotome, to our knowledge. Hence, we propose a depletion model (Fig. 7). Our work shows a correlation between decreased sclerotome in *smb* fish and a later lack of dorsal fin. The decreased sclerotome does not appear to affect early derivatives such as fibroblasts and tendons. However, by the time the vertebral spines, anal and dorsal fins form from that initial sclerotome population, this smaller pool has been depleted and there are not enough cells to form the dorsal fin (Fig. 7). Regardless, it is striking that a change in an embryonic tissue at 24 hpf may lead to a phenotype not overtly detectable until 2 wpf.

As with many craniofacial zebrafish mutants (Nichols et al., 2013; Noden, 1983; Schilling et al., 1996; Sucharov et al., 2019), we propose that the musculature defects seen in *smb* are secondary. There are no skeletal structures for muscles to attach to in the dorsal fin; in the anal fin, the disorganized radials may lead to incorrect muscle attachment.

The *smb* transgenic insertion does not interfere with any gene coding region. Instead, we hypothesize its ectopic regulatory sequences most likely dysregulate the expression of an endogenous nearby gene or genes. It is also possible this dysregulation targets different genes at various developmental windows. Focused RNA-sequencing of sclerotome cells during the expansion state (24 hpf) is needed to better understand the molecular genetics of this mutant and how the transgene affects gene expression at this sensitive window. The new regulatory sequences may alter the neighboring genomic landscape as well. It would be interesting to ‘bash’ the integrated transgene, i.e., delete specific regions, to test if the *sox10* regulatory sequences introduced by the transgene are causative of the phenotype. Further, ATAC-seq and HiC sequencing would help better understand the interaction between the exogenous transgene and the endogenous genome. These experiments may shed light on any differential chromatin accessibility in wild types and mutants as well as identify regions of the genome contacting the *smb* transgene. Layering these assays of gene expression, chromatin accessibility, and topology on top of each other will build a coherent model for how the *smb* insertion affects chr17 and leads to the absent dorsal fin phenotype we observe.

Though many different tissues and cell types, including the axial skeleton, have been traced back to the sclerotome of vertebrates, the source of the median fins has remained mysterious. This is likely because many other vertebrate models do not develop median fins. For many decades, these fins were believed to originate from trunk neural crest. Lee et al. (2013b) showed that the fin ray osteoblasts of the zebrafish median fins are derived from the paraxial mesoderm, but did not further investigate from which subcompartment these cells arise. Here, we definitively show for the first time that the sclerotome is the source of the external dermal rays and endochondral radials of the dorsal and anal fins.

Why do fish possess two separate sclerotome domains rather than one single ventral domain, as in mouse and chick? It is possible that the dorsal sclerotome domain in zebrafish is required to generate dorsal structures unique to basal vertebrates. Through cell tracing in *nkx3*.*1^NTR-mCherry^* transgenic fish, Ma et al. (2018) showed that some cells from the dorsal sclerotome domain migrate dorsally and contribute to fin mesenchymal cells in the fin fold; these cells remain in the adult fin as inter-ray fibroblasts (Lee et al., 2013b). Therefore, the dorsal sclerotome might be a unique feature for finned species, including those that just form fin folds but not bony fins, like amphibians. Since mice and chick do not have dorsal fins or fin folds, the dorsal domain is not required and was therefore disposed of during evolution. Consistent with this idea, we observe a reduced dorsal sclerotome in our *smb* mutants, which lack a dorsal fin. In *smb* fish with partial dorsal fin loss, it is possible that these individuals have a less severe depletion in their dorsal sclerotome volume and thus there are enough progenitors to form some skeletal structures in the dorsal fin. Unfortunately, we cannot probe sclerotome volume of individuals and grow these to analyze their corresponding fins as HCR requires fixed tissues and live imaging of the *nxk3.1:Gal4* line is unfeasible due to the *smb* insertional mutant having *Gal4* in its transgenic sequence.

The sclerotome is derived from the somites, which evolved before the origin of vertebrates in an ancestral invertebrate chordate (Delsuc et al., 2008). In the cephalochordate amphioxus, only a ventral sclerotome compartment is present; these cells later migrate medially and dorsally along midline structures as development ensues (Mansfield et al., 2015). Although these invertebrates do not develop a dorsal fin skeleton, a medial segmental structure termed the ‘fin box’ grows dorsally along the midline. In amphioxus, these structures are derived from the ‘external cell layer’ of the somites, not the sclerotome (Mansfield et al., 2015), so the ontogeny of these structures is different from true median fins. However, it is possible that true fins evolved when the dorsal sclerotome domain evolved in fish. In this scenario, the dorsal sclerotome could populate the preexisting fin box and deploy a skeletal differentiation program. Future experiments addressing changes in somitic mesenchyme across evolution specifically analyzing the appearance or disappearance of a bipartite sclerotome could address the origin of true median fins.

Finally, we caution researchers regarding potential off-target effects of transgenesis. In our case, it is possible we have created a new regulatory sequence that leads to the loss of an appendage without affecting other organs or viability. Unknowingly, the transgenes we insert might produce changes in the surrounding genome (Liu and Liu, 2012). After all, it is changes in regulatory sequences that foster the evolution of developmental mechanisms and major shifts in organismal form (Shubin et al., 2009; Wagner, 2014).

## MATERIALS AND METHODS

### Zebrafish strains and husbandry

All zebrafish experiments used the AB strain. Animals were maintained and staged according to established protocols (Fishman et al., 1997; Kimmel et al., 1995; Parichy et al., 2009). All our work with zebrafish has been approved by the University of Colorado Institutional Animal Care and Use Committee (IACUC) (protocol number for animal research: 00188), and the Animal Care Committee of the University of Calgary (#AC21-0102). The following zebrafish lines have been previously reported: *Tg*(*fli1a:EGFP*)*^y1^* (Lawson et al., 2002), *Tg*(*tbx6:CRE*)*^sq6Tg^* (Lee et al., 2013b), *sox9a:EGFP* (*sox9^azc81Tg^*) (Eames et al., 2013), *Tg(-3.5ubb:LOXP-EGFP-LOXP-mCherry)^cz1701Tg^* (Mosimann et al., 2011), *Tg*(*scxa:mCherry*)*^fb301Tg^* (McGurk et al., 2017), *Tg*(*alx4a:DsRed2*)*^pd52^* (Nachtrab et al., 2013), *TgBAC(hand2:EGFP)* (Yin et al., 2010), *Tg*(*UAS:Crimson,myl7:EGFP*)*^b1229Tg^* (Nichols et al., 2013), *Tg(her6:mCherry)^sd64Tg^* (Kraus et al., 2022), *TgBAC(nkx3-1:GAL4-VP16)^ca101Tg^* (Ma et al., 2018), *Tg(UAS:Cre-ERT2)^ca105Tg^* (Sharma et al., 2019), and *lof^dt2^* (Tresnake, 1981). The *smb* mutant, *smb^Tg(sox10:Gal4)co3021^*, is a transgenic construct insertion not known to create an allele of any gene.

### Cartilage and bone staining and imaging

Adult animals were fixed overnight in 2% paraformaldehyde (PFA) and were stained with Alcian Blue and Alizarin Red (modified from Walker and Kimmel, 2007). Briefly, fixed animals were washed with 100 mM Tris (pH 7.5), 10 mM MgCl_2_ for 1 h and stained with 0.01% Alcian Blue in 10 mM MgCl_2_ (pH 7.5) overnight at room temperature. Samples were then re-hydrated in a series of washes: 80% ethanol, 100 mM Tris (pH 7.5), 10 mM MgCl_2_; 50% ethanol, 100 mM Tris (pH 7.5), 10 mM MgCl_2_; 25% ethanol, 100 mM Tris (pH 7.5); 0.5% KOH each for 30 min. Fish were bleached with 3% H_2_O_2_; 0.5% KOH until the pigment was gone in the body and eyes became light brown. We then digested and cleared the samples with 35% saturated (Sat.) NaBO_4_ for 30 min and 1% Trypsin in 35% Sat. NaBO_4_ overnight at room temperature. Samples were washed in 10% glycerol, 0.5% KOH for 1 h. Fish were stained with 0.02% Alizarin Red (pH 7.5) overnight at room temperature and washed again in 10% glycerol, 0.5% KOH overnight. For late larval stage animals (<10 mm SL) a larval staining protocol was used as previously described (Brooks and Nichols, 2017; Walker and Kimmel, 2007). For whole body skeletal imaging, we used a SteREO Discovery V8 Zeiss scope connected to an Axiocam 105Color camera, then we stitched tiles using Adobe Photoshop.

### Phalloidin staining

Fish were euthanized and fixed in 4% PFA/PBS for 4 h at room temperature. Samples were washed four times in PBS/0.1% Triton X-100 for 30 min each followed by an incubation in PBST+1:50 Phalloidin for 45 min. Extensive PBS washes were performed to stop the staining.

### Live imaging

Fluorescent images were captured using a Leica DMi8 microscope equipped with an Andor Dragonfly 301 spinning disk confocal system. Images were analyzed using Imaris and FIJI image analysis tools. Acquisition parameters and fluorescence adjustments were applied linearly and equally to all samples. For still images, we used 4% tricaine in E2 to anesthetize the fish.

### Embryo injection and transgenesis

We generated the *sox10:GAL4VP16* construct that produced the *smb* insertion with the 3136 nucleotides upstream of the *sox10* initiating methionine codon and the Tol2 kit (Kwan et al., 2007). Plasmid DNA was prepared using the HiSpeed Plasmid Midi Kit (Qiagen). Transposase mRNA was synthesized from SmaI-linearized plasmid template transcribed with T3 RNA polymerase (Ambion). Embryos were injected with 30 ng/μl plasmid DNA and 30 ng/μl Tol2 mRNA diluted in Phenol Red using a microinjector (Applied Scientific Instrumentations).

### Cloning the genomic locus of the *smb* insertion

Genomic DNA was isolated from the caudal fin of an *smb* adult genotyped via *UAS:E2Crimson* activity and from one non-fluorescent sibling adult (Qiagen DNeasy). Then 1 μg gDNA from each of these samples was digested with AluI in 40 μl at 37°C for 3 h, and 0.5 μg of digested gDNA was ligated with 4 μl T4 DNA ligase (Epicentre) in 500 μl overnight at 16°C. The reaction was concentrated using a Zymo-5 column (Zymo Research) and resuspended in 20 μl for use as PCR template. Inverse PCR was carried out according to published protocols for cloning Tol2 insertion sites from the zebrafish genome (Kawakami et al., 2004; Kotani et al., 2006). First-round PCR primers were either [Tol2-3′invf2+Tol2-3′/r1] or [Tol2-3′invf1+Tol2-3′invr1], while second-round PCR primers were either [Tol2-3′/f2+Tol2-3′invr2] or [Tol2-3′invf2+Tol2-3′invr2] (Kawakami et al., 2004; Kotani et al., 2006). No PCR products were obtained from non-transgenic fish. Many clones from products of the different PCR primer pairs were sequenced from each transgenic fish, and sequences BLASTed to the same location in the zebrafish genome.

### Genotyping assays

To genotype *smb* heterozygotes, we PCR-amplified across the *sox10:Gal4* transgene (Fw: 5′-CGACACTCCCAGTTGTTCTT-3′; Rv: 5′-AGCCTCAGTGTTTGTAGGTTT-3′). The product is a 675 bp amplicon in *smb* while wild types do not amplify a product due to the absence of transgenic sequence. To genotype for *smb* homozygotes, we used a 25 bp SSLP that is tightly linked to the insertion site and only amplifies in the wild-type allele but not when the *smb* insertion is present. We crossed two heterozygous *smb* parents each with a different allele of the SSLP on their wild-type chromosome. These different wild-type chromosomes produce either a 201 bp or a 226 amplicon with the following primers: Fw: 5′-GATTCAGACATTGCCCTGTAGT-3′; Rv: 5′-TAGATGGACCCTTACGAACCT-3′. In this cross, homozygous wild-type offspring are heterozygous for the SSLP producing both 226 and 201 bp amplicons. Homozygous *smb* offspring do not amplify, and *smb* heterozygous animals produce either the 226 or the 201 band.

### Hybridization chain reaction

*In situ* HCR v10 (Choi et al., 2014) with standard probes was performed using the detailed whole-mount zebrafish embryos and larvae protocol available at Molecular Instruments.com. Experiments were performed in fish at different stages. Briefly, after fixation overnight at 4°C, embryos were dehydrated and permeabilized with a series of 100% methanol washes and stored overnight. Rehydration with a series of graded methanol in phosphate-buffered saline with 0.1% Tween 20 (PBST) was then performed. Samples were pre-hybridized for 30 min at 37°C with Molecular Instrument-provided buffer. Later, samples were hybridized in probe solution containing 2 pmol of each probe and left incubating overnight at 37°C. After two 5× SSCT washes, samples were amplified in Molecular Instruments-provided Amplification Buffer containing 30 pmol of snap-cooled hairpin pairs. Samples were left overnight in the dark at room temperature. The next day, a series of washes in 5× SSCT was performed to remove excess hairpins. Finally, the samples were stored at 4°C protected from light before imaging.

### Surface rendering analysis

*Z*-stacks of HCR data were analyzed using Imaris 9.2, and display adjustment settings were uniformly set across all samples. After generating a surface scene, a region of interest was set for the ten most proximal somites to the vent (five anterior and five posterior). With a surface detail of 2.41 and the smooth feature selected, the threshold (absolute intensity) was set manually to best represent the signal in the sample (uniform across samples). The sum of volume was used to assess the HCR-FISH signals.

### Statistical analyses

For HCR analyses, we used unpaired two-tailed Student's *t*-test. We used Chi-square test for analysis of expected versus observed Mendelian ratios. For all other analyses comparing more than two groups, we used one-way ANOVA. All analysis were performed in GraphPad (Prism). Error bars represent s.e.m. For significance of penetrance tables, we used Fisher's exact test.

### Cre-mediated lineage tracing

Transgenic *nkx3.1:Gal4; UAS:Cre-ERT2; ubi:Switch* embryos were treated with 10 μM 4-OHT from 1 to 2 dpf. After treatment, embryos were washed three times and recovered in fish water for analysis at the relevant developmental stages.

## Supplementary Material



10.1242/develop.203025_sup1 Supplementary information
