# Supplementary material for: The sclerotome is the source of the dorsal and anal fin skeleton and its expansion is required for median fin development
Source: Development. 2024 Dec 13;151(24):dev203025. doi: 10.1242/dev.203025 (PMC11664171; doi:10.1242/dev.203025)
Supplement: Supplementary information [file develop-151-203025-s1.pdf]

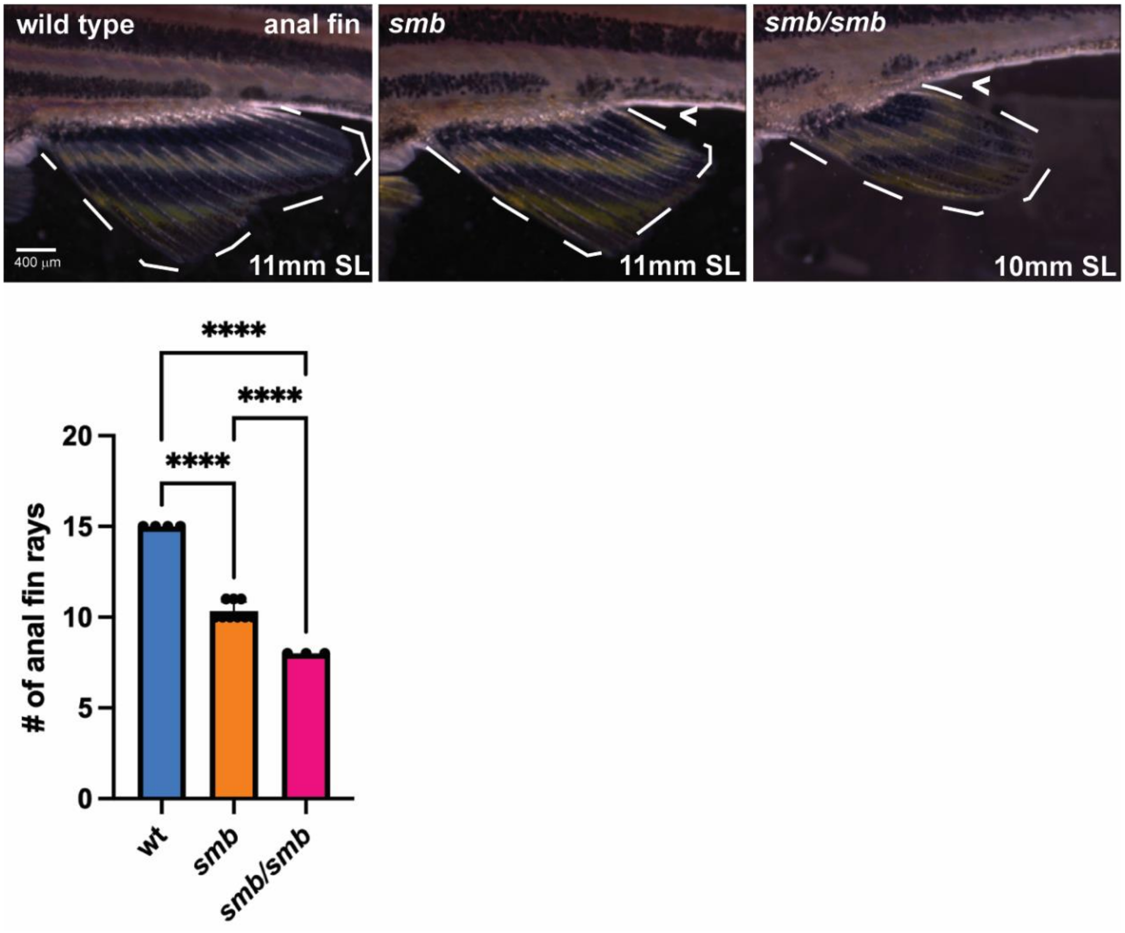

Fig. S1. Homozygous *smb* mutants have further reduced anal fins compared with heterozygotes. Adult anal fins from wild types, *smb* heterozygous (*smb*) and *smb* homozygous (*smb/smb*) mutants were imaged with transmitted light. The number of anal fin rays was quantified for each genotype. A one-way ANOVA was performed for statistical analysis, with **** indicating a p value < 0.001. Carets indicate reduction in the anal fins of mutants.

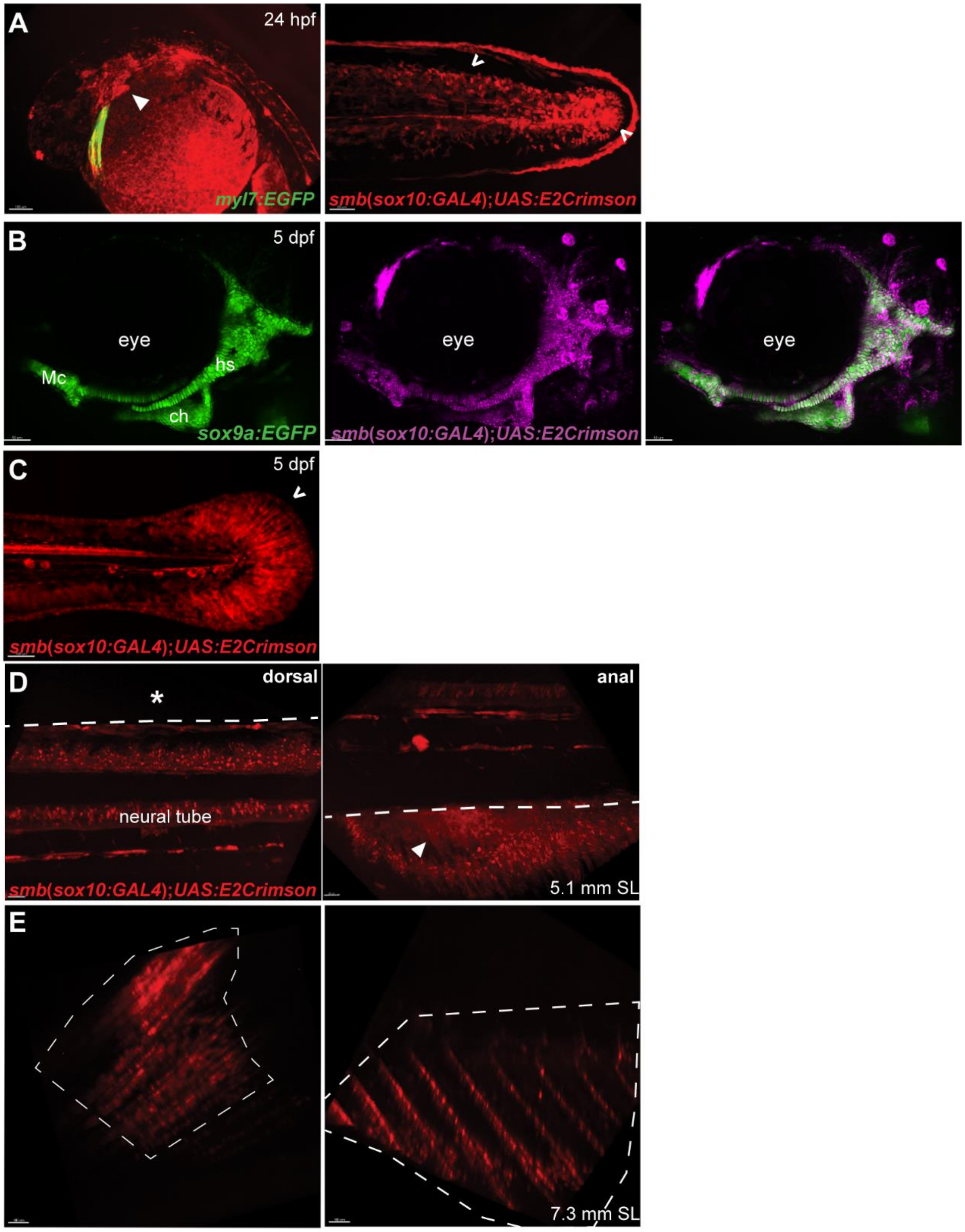

Fig. S2. The *smb*(*sox10:Gal4*) transgene insertion labels mesoderm derivatives.

A: *smb*(*sox10:Gal4*) heterozygous fish were crossed to the *UAS:E2Crimson* reporter fish, and double transgenic offspring were imaged by confocal microscopy at 24 hpf. Arrowhead indicates faithful labeling of the pharyngeal arches by the *smb* transgene, as seen by *Gal4*

activity and E2 Crimson fluorescence. Carets indicate mesoderm-derived cells at 24 hpf labeled by this transgene. Bar= 100 µm. **B:** Heterozygous *smb*(*sox10:Gal4*)*;UAS:E2Crimson* fish were crossed to *sox9a:EGFP* fish , and triple transgenic fish were imaged by confocal microscopy at 5 dpf. EGFP expression is shown in craniofacial chondrocytes of the Meckel's cartilage (Mc), hyosymplectic (hs) cartilage, and ceratohyal (ch) where *Gal4* is also expressed. Bar= 50 µm **C:** Caret indicates labeling of caudal fin fold fibroblasts by *smb*(*sox10:Gal4*);*UAS:E2Crimson* (n=3/3 mesodermal expression). Bar= 100 µm. **D:** Heterozygous *smb*(*sox10:Gal4*) fish were crossed to the *UAS:E2Crimson* reporter, and double transgenic fish were imaged at 5.1mm SL by confocal microscopy. Asterisk indicates absent dorsal fin mesenchyme, arrowhead indicates *Gal4* activity in the developing anal fin bud (n=3/3 Crimson-positive mesenchyme). Bar= 40 µm. **E:** Animals stably carrying an independent insertion of the *sox10:Gal4* construct were crossed to the *UAS:E2Crimson* reporter, and double transgenic fish were imaged by confocal microscopy as adults. Outline indicates median fins that develop in this independent integration of the transgene construct (n=3/3 new stable integrants with median fins). Bar= 100 µm.

Development • Supplementary information

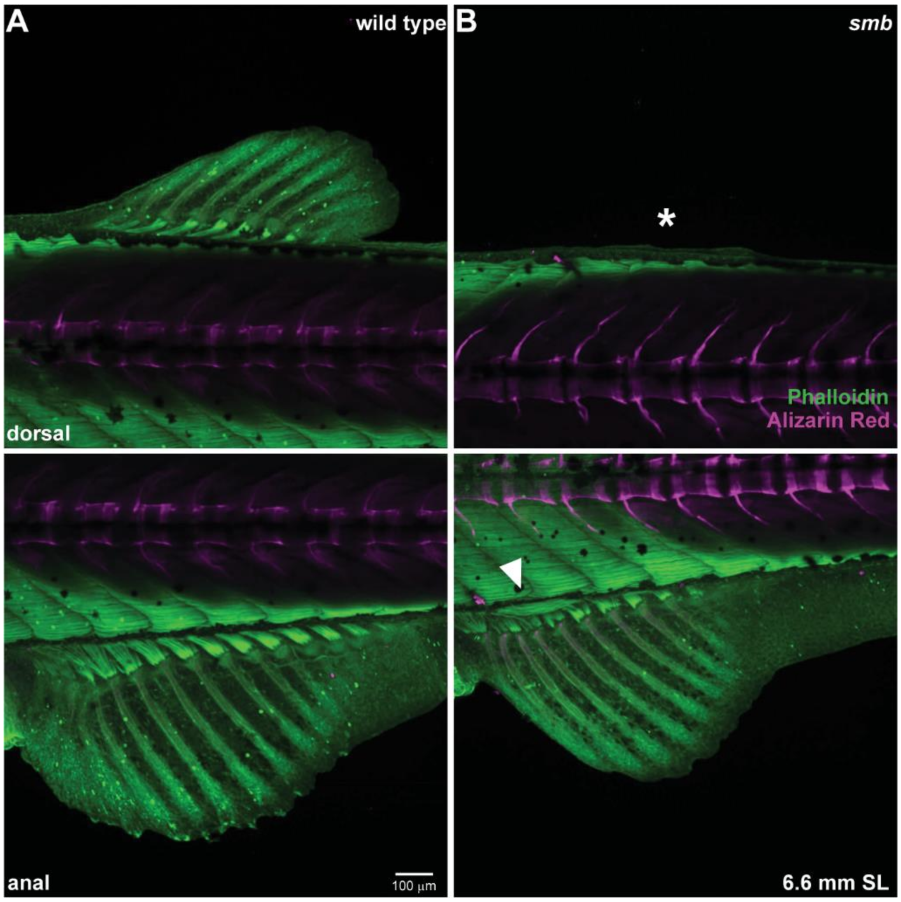

Fig. S3. *smb* mutants develop disorganized anal fin muscles. A: Median fin muscles of wild-type fish stained with phalloidin and mineralized bone stained with Alizarin Red were imaged by confocal microscopy. **B:** In *smb* heterozygous mutants, asterisk indicates absent dorsal fin with no muscles. Arrowhead indicates disorganized anal fin muscle attachments (n=3/3 mutants with disorganized muscle fibers). Variable staining of phalloidin and Alizarin Red is due to specimen orientation.

Development • Supplementary information

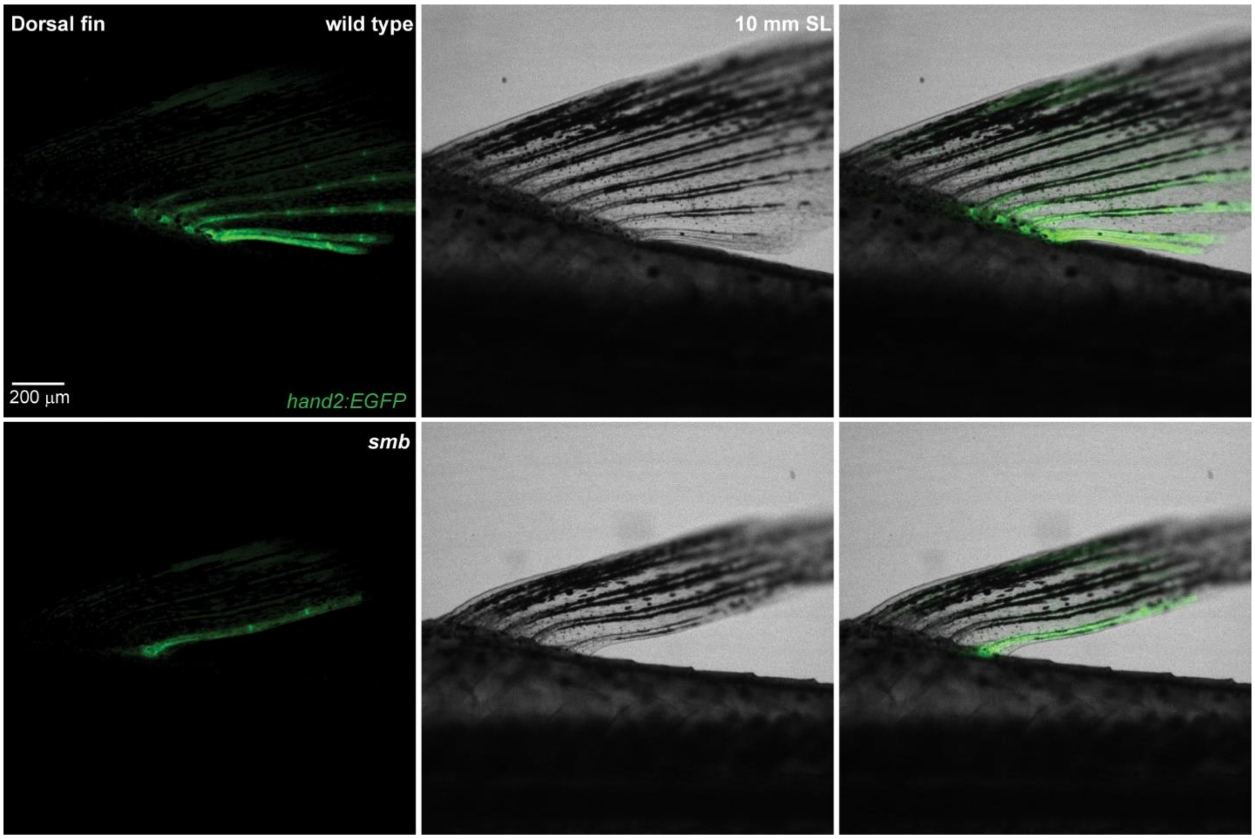

Fig. S4. Posterior-identity skeletal structures are lost in the partial dorsal fins of *smb* mutants. Posterior dorsal fin rays labeled with *hand2:EGFP* were live imaged in wild-type and *smb* heterozygous fish with partial fins (wt=3, *smb*=2).

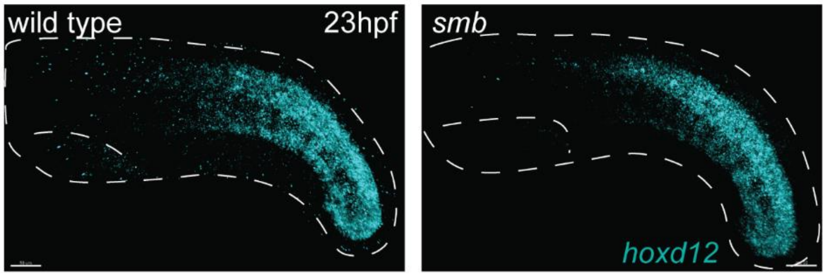

Fig. S5. *hoxd12* expression is unchanged in *smb* mutants. *hoxd12* transcripts were fluorescently labeled with HCR in wild-type (n=7) and *smb* heterozygous (n=4) embryos at 23 hpf and imaged by confocal microscopy. *smb* mutants had no overt differences in location or intensity of expression compared to wild-type siblings. Bar= 50 µm.

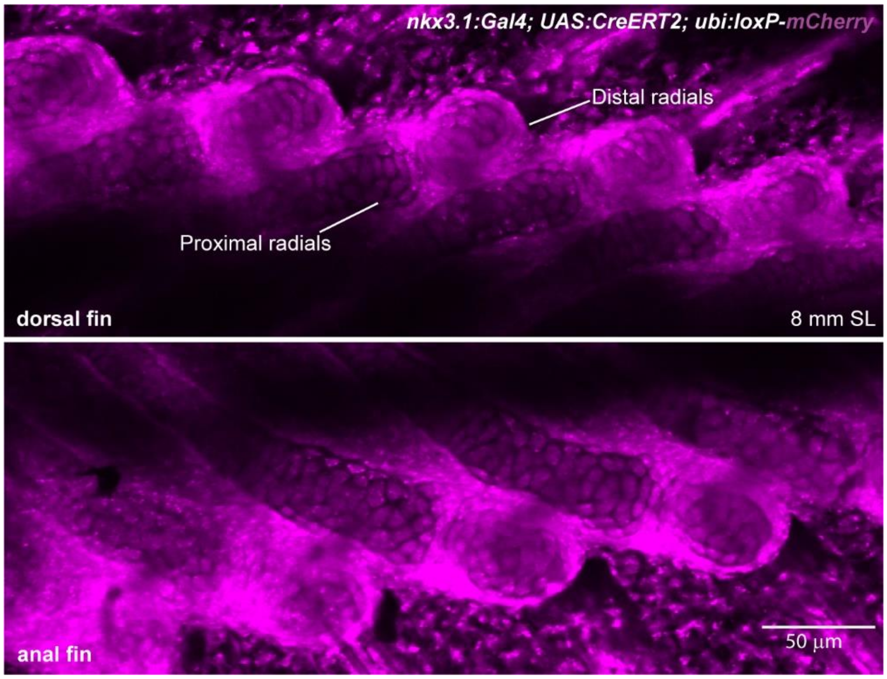

Fig. S6. Endoskeleton of the dorsal and anal fins is derived from the sclerotome. Enlarged images from Fig. 5B of sclerotome-derived, Cre-recombined, mCherry expressing chondrocytes in the proximal and distal radials of the dorsal and anal fins.

Development • Supplementary information

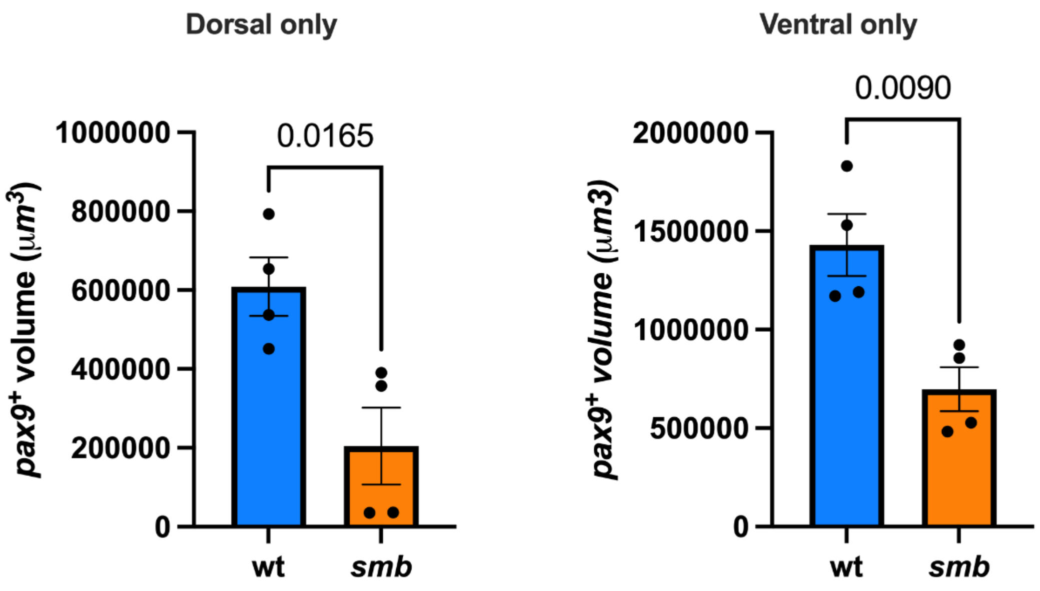

Fig. S7. Dorsal and ventral sclerotome domains are reduced in *smb* heterozygous mutants at 24 hpf. Dorsal and ventral domains from Figure 6A, 24hpf timepoint were quantified and analyzed independently to assess individual domain differences.

Development • Supplementary information

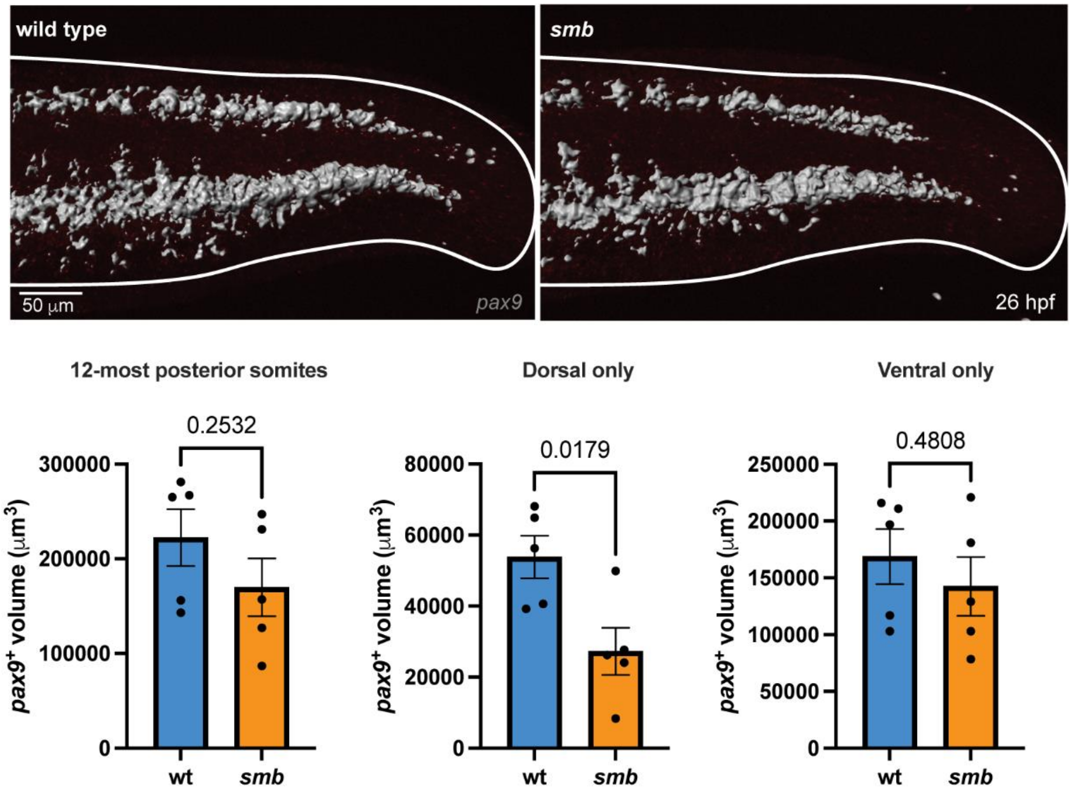

Fig. S8. Only the dorsal sclerotome domain of *smb* mutants is significantly reduced later in development. *pax9* transcripts were fluorescently labeled with HCR in wild-type and *smb* heterozygous embryos at 26 hpf and imaged by confocal microscopy. Grey indicates the *pax9* positive domain used to quantify volume using Imaris surface rendering. *pax9* expression was quantified by Imaris (n= 5 per genotype). The volume was quantified for both domains (t-test; p=0.2532), dorsal only (t-test; p=0.0179), and ventral only (t-test; p=0.4808).

Development • Supplementary information

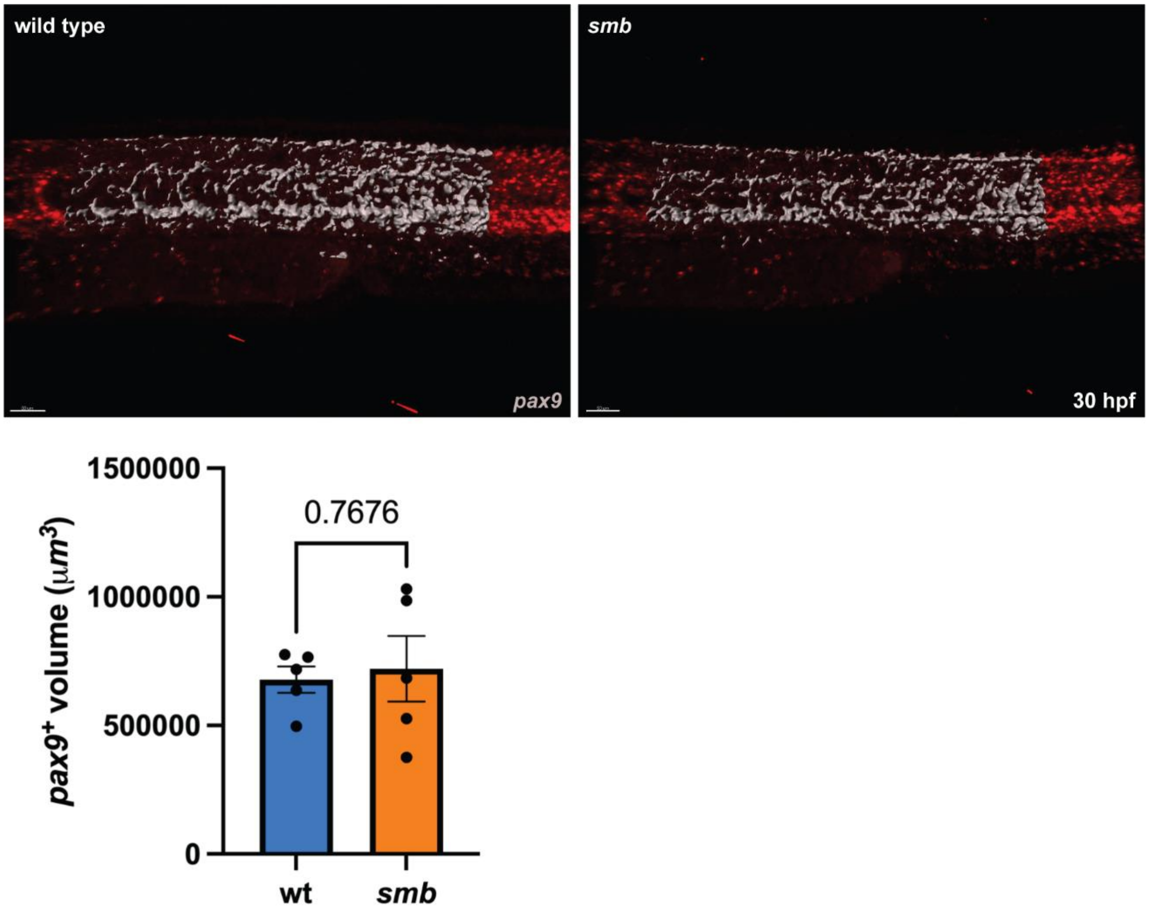

Fig. S9. *pax9* expression is unchanged at 30hpf in *smb* mutants. *pax9* transcripts were fluorescently labeled with HCR in wild-type and *smb* heterozygous embryos at 30 hpf and imaged by confocal microscopy (n= 5 per genotype). Grey indicates the *pax9* positive domain used to quantify volume using Imaris surface rendering (t-test; p=0.7676). Bar= 50 50 µm.

Development • Supplementary information
