# Supplementary material for: The sclerotome is the source of the dorsal and anal fin skeleton and its expansion is required for median fin development
Source: Development. 2024 Dec 13;151(24):dev203025. doi: 10.1242/dev.203025 (PMC11664171; doi:10.1242/dev.203025)
Supplement: Supplementary file 1 [file develop-151-203025_review_history.pdf]

The sclerotome is the source of the dorsal and anal fin skeleton and its expansion is required for median fin development

Raisa Bailon-Zambrano, Margaret K. Keating, Emily C. Sales, Abigail R. Nichols, Grace E. Gustafson, Colette A Hopkins, Katrinka M. Kocha, Peng Huang, Lindsey Barske and James T. Nichols
DOI: 10.1242/dev.203025

Editor: Steve Wilson

Review timeline

Original submission:	2 May 2024
Editorial decision:	19 June 2024
First revision received:	17 September 2024
Editorial decision:	25 October 2024
Second revision received:	1 November 2024
Accepted:	13 November 2024

Original submission

First decision letter

MS ID#: DEVELOP/2024/203025

MS TITLE: The sclerotome is the source of the dorsal and anal fin skeleton and its expansion is required for median fin development

AUTHORS: Raisa Bailon-Zambrano, Margaret Keating, Emily C. Sales, Abigail R. Nichols, Grace E. Gustafson, Katrinka M. Kocha, Peng Huang, Lindsey Barske, and James T. Nichols

I have now received all the referees' reports on the above manuscript, and have reached a decision. The referees' comments are appended below, or you can access them online: please go to BenchPress and click on the 'Manuscripts with Decisions' queue in the Author Area.

As you will see, the referees express considerable interest in your work, but have some criticisms and many detailed suggestions for improving the study and manuscript. If you are able to revise the manuscript along the lines suggested, I will be happy receive a revised version of the manuscript. Please also note that Development will normally permit only one round of major revision. If it would be helpful, you are welcome to contact us to discuss your revision in greater detail. Please send us a point-by-point response indicating your plans for addressing the referees' comments, and we will look over this and provide further guidance.

Please attend to all of the reviewers' comments and ensure that you clearly highlight all changes made in the revised manuscript. Please avoid using 'Tracked changes' in Word files as these are lost in PDF conversion. I should be grateful if you would also provide a point-by-point response detailing how you have dealt with the points raised by the reviewers in the 'Response to Reviewers' box. If you do not agree with any of their criticisms or suggestions please explain clearly why this is so.

Reviewer 1

Advance summary and potential significance to field

© 2024. Published by The Company of Biologists under the terms of the Creative Commons Attribution License (https://creativecommons.org/licenses/by/4.0/).

The manuscript by Bailon-Zambrano et al. details new important findings on the derivation and specification of medial fins. While much is known concerning paired fin development and specification due to the homology to limbs in tetrapods (and us), there is little data on how the medial fins are specified during development and evolution. While the immediate impact and need for an understanding of medial fin development is not always appreciated, these fins are historically ancestral to paired fin and their development sets the context for paired fin evolution, in essence why certain patterning and pathways are essential for appendage formation in general. Thus, knowing the foundational mechanisms of paired fin development will be important to illuminate how and why fin/limb appendages arise and diversify. The authors capitalize on a mutant zebrafish line identified as having loss of dorsal fins as adults, with varied reduction in anal fin formation. Such a 'smoothback' phenotype (smb) has been seen in isolated lineages in nature, as well as in domestication of fancy fish strains. The investigation of this line provides inroads into understanding this variation. The authors provide elegant anatomical analysis, describing the affected structures and tissues in the smb mutant. Importantly, they show that the medial fins are derived from sclerotome, something that was not shown previously. They show that the effect of the mutation is due to a reduction of condensation of these cells that presage the development of the fins. By yet undefined mechanisms, they define a scleorotome depletion model over development to detail the specificity exposed in the smb mutant. This descriptive analysis is new and important in understanding the foundations of how fins may have evolved. However, little mechanistic data is provided as to how this phenotype is regulated by the locus. This limits a broader integration of the mutant phenotype in the extended Developmental community. More information on the differential shift in transcriptional regulation and what pathways are either perturbed or ectopically activated will provide an important context towards understanding developmental mechanism. Further, integrating the action of the mutant with extended epistasis analysis of mutants affecting dorsal fin formation or sclerotome function may be illustrative of 'how' (e.g. function).

Comments for the author

In assessing revision, it would be helpful to comment on a couple of points from the material presented.

In efforts to show position-dependent effect of the smb mutant, the authors use repeated insertional analysis to show that the effect was unique to smb. However, the authors use the fact that they recover independent lines having similar expression in forming fins – however without a loss of fin growth. This is confusing as the model posed by the authors for the causal effect of the insertion is that it leads to mis or enhanced expression within early progenitor populations. This effect would be thus specific to the insertion site. The finding of similar fin expression in independent lines that are presumed unlinked due to lack of phenotype is confounding, especially as the Sox10 enhancer locus does not normally drive expression in these tissues. Some clarification of this fact/statement would be helpful.

The epistasis analysis with longfin highlights classic and really impactful approaches in the use of mutants to discern mechanisms. The longfin analysis places the action of smb prior to growth signals. However, as the hypothesis centers on early populations, it would be useful and more interesting if epistasis analysis can be done on mutants that affect sclerotomal specification to more precisely detail smb action.

The deduction, or inference of potential neural crest contribution to the caudal fin mesenchyme is not justified/supported. The experiment with Sox10 is not lineage tracing of NCC, but most likely leaky, or pleiotropic expression of Sox10 transgene. The authors would need to do classic lineage analysis, similar to the sclerotome analysis done in this manuscript, to make this connection. The variegation in labeling the authors see is most likely incomplete labeling or silencing of the transgene in mesenchymal clones leading to the formation of the caudal fin anlagen.

It would also smooth the logic to have F2 dose-dependency analysis moved forward in the results to when the mutant is initially characterized. This will focus the logic of the paper.

© 2024. Published by The Company of Biologists under the terms of the Creative Commons Attribution License (https://creativecommons.org/licenses/by/4.0/).

Lastly, while certain parts of the paper were written very well, the impact and integration of these findings were not made clear. This resulted in a disconnected feel of the narrative. What is known of sclerotome contribution, or embryonic lineage of medial fin development overall; why is this important; what does it imply for formation of paired fins and plasticity? Could an evolutionary model be made integrating somitic mesodermal contributions to medial fin formation? In part extending from even from chordates and anamniotes (such as in PMID: 26052418) could be very informative and might lead to experiments to address changes/evolution of somitic mesenchyme contribution

Reviewer 2

Advance summary and potential significance to field

The manuscript by Raisa Bailon-Zambrano et al. entitled "The sclerotome is the source of the dorsal and anal fin skeleton and its expansion is required for median fin development" is a significant addition to the field of fin develoment. Through analysis of the newly generated sox10:Gal4 transgenic insertion mutant "smoothback", Bailon-Zambrano et al. are able to identify the sclerotome as the mesodermal subcompartment that provides the progenitor cells for the median fin exo- but also endoskeleton. Analysis of this mutant also provides an important inroad to the question of median fin positioning and induction.

Comments for the author

Results
smoothback (smb) is a dominant mutant with median fin phenotypes caused by a transgenic insertion I encourage revision and publication of this interesting manuscript upon addressing the following points:

p.7 1st paragraph: after screening for GAL4 activity in F1 integrants
The description of the experimental proceedings of screening and identification of the smb mutant is difficult to understend. Please indicate the stage and method you used to identify and screen for GAL4 activity in "stable germline-transmitting integrants" (F1), and which subsequent crossing scheme led to F2 families.
I also don't understand why "the dorsal fin loss is only partial in some families"? Do you refer to individuals within the family showing the smb phenotype?
Also explicitly state, in the text and also the figure legends, whether you show a heterozygous or homozygous smb mutant (c.f. Figs.1C and S1)

p.8 1st paragraph: linkage of smb-phenotype and transgenic insertion: here you propose that the transgene "is either causative of the loss of fin phenotype or tightly genetically linked". This implies that the transgene disrupts (or is positioned close to) a genetic regulatory element that is necessary for fin positioning or fin induction.
This is an extremely interesting implication of the mechanism affected in the mutant that you cannot ignore. I suggest, you produce a crispr/CAS deletion of the non-coding region around the insert site of a wild-type chr17:46,219,972 (see next comment) to demonstrate lack of the dorsal fin in a second genetic allele at this site (a deletion allele). This may be a one-generation experiment with sufficiently high gRNA concentration as a proof of principle experiment for the matter of this manuscript.

p.8 The smb-causing transgene drives Gal4 in ectopic tissues and does not disrupt a coding sequence.
2nd paragraph: "we crossed a smb mutant to UAS:E2Crimson fish to visualize Gal4 activity": pharyngeal arch labelling: Fig.S2b clearly shows labelling of the hyosymplectic and the ceratohyal, the two elements of the hyoid arch, to a knowledgeable observer. All others may not understand the significance of the image. If you could provide a higher power view of the image, that shows the round structure of the cartilage cells within these red elements, the figure would convince readers that head cartilages are stained.
Fig.S2 a&b,c,d: staining in the paraxial mesoderm and mesenchyme of the median fin fold: instead of saying "We conclude that the smb phenotype is genomic integration site-dependent and is not

© 2024. Published by The Company of Biologists under the terms of the Creative Commons Attribution License (https://creativecommons.org/licenses/by/4.0/).

strictly due to Gal4 protein expression in fin mesenchyme. These results motivate the hypothesis that transgene integration into a specific genomic locus affects endogenous gene expression or gene function or both to cause the smb phenotype." You should explicitly state in the text that aside from the labelling of the hyoid arch cartilages, the transgene provides two functions: 1. It can act as an enhancer trap, staining mesodermal cells in the trunk and median fins and 2. at the smb genomic integration site, it mutates a dorsal-fin specific regulatory element which leads to the smb phenotype (hence the above suggestion of creating a second allele by crispr/CAS deletion, to verify this assertion)

p.9/10 The dorsal fin skeleton fails to develop and anal fin skeletal elements are disorganized in smoothback mutants
p.10 second paragraph: the sox9a:EGFP-line labels cartilaginous elements, i.e. the endoskeletal radials of the dorsal fin. However, you need to show that, at an earlier stage, the dorsal fin bud/field mesenchyme does not accumulate at its regular position above somites 11 - 15, i.e. if the mesenchyme fails to accumulate, there is no sign or fin induction! You can visualize this by "Nomarsky" optics of the dorsal fin fold of 2 weeks old fish. Bleach pigment if it is in you way or use a pigment mutant so that you can see the initial undifferentiated "bud" mesenchyme. The question is how a lack of fin mesenchyme aggregation comes about. In any case, it is central to the interpretation of the mechanism causing the smb phenotype, viz. axial patterning/fin induction vs. a late failure of fin bud growth/differentiation. It is important that you report evidence that favors one vs. the other cause of the smb phenotype. see next comment

p. 9/10 in comparison to p.6, introduction: in your introduction you introduce the t-box gene eomesa as a necessary factor for dorsal fin formation in zebrafish and carp. Even though this gene was found to be necessary for dorsal fin develoment, its expression pattern has not been reported. Two other t-box transcription factors, tbx5 and tbx4 are necessary for paired fin/limb formation. They are the earliest markers of pectoral and pelvic fin formation in zebrafish, respectively.
Is eomesa likewise an early marker of the dorsal fin mesenchyme? It would be extremely rewarding if you could undertake this in-situ hybridization, because it would demonstrate the activity of another t-box gene during early fin development and would add to the (ancient) homology argument of unpaired and paired fins the developmental perspective of t-boxes regulating the developmental induction of all appendages!
In addition, you could argue that smb fish don't show early signs of dorsal fin induction instead of arguing smb don't show cartilage radial differentiation in the fin mesenchyme, which is too late.

p11/12: smb mutants do not establish the dorsal fin bud
p12 first paragraph: "These results motivate the hypothesis that smb affects one of the earliest reported steps in median fin formation":
As pointed out above, you should be able to show that "the earliest steps in median fin formation" is affected in smb mutants and not leave it to a hypothesis, open to discussion.

p.13 The sclerotome is the source of the median fin skeleton this is a strong section in the manuscript! May I encourage you to provide a higher magnification image of Fig.4B such that the reader can actually appreciate the condrocytic distal and proximal radials at the ends of the arrowheads. (You might have to cut away the muscles) There is only little mention in the literature that the endoskeletal radials are of sclerotomal origin, though they were always assumed to be. The neural crest hypothesis also only explicitly addressed the origin of the lepidotichs. Your lineage tracing image could show that both skeletal compartments originate from one progenitor population.

p14: Sclerotome expansion is decreased in smb mutants
please explain what we see in Fig.5 There is a paraxial region of expression lateral to the vertebral column? but there also is a detached "dorsal expression domain". Are these cells the ones that give rise to the dorsal fin? Which pax9 cells did you count in your quantification?

p15/10: Early sclerotome derivatives remain unaffected in the smb mutant
You conclude that "that derivatives of the sclerotome that emerge at early larval stages are unaffected in the smb mutant, while the later forming structures like the median fins are more sensitive".

© 2024. Published by The Company of Biologists under the terms of the Creative Commons Attribution License (https://creativecommons.org/licenses/by/4.0/).

In this context, it may be interesting to probe for an anterior-posterior axial patterning defect in the smb mutant while mesoderm segmentation per se might be correct. In the paper of Frank van der Hoeven et al. 1995. Mechanisms of Development 54 9-21. the authors point out that the mesodermal axial expression patterns of hoxd genes show the expected collinear expression. Especially hoxd11/hoxd12 show an anterior border of their expression domains at somite 11 and 17, respectively, which marks the region of the anterior margin of dorsal and anal fins. Again, an in situ hybridization with these markers around 22hpf should show if smb shows an a/p patterning defect that would consequently lead to a lack of dorsal fin induction vs. a fin induction defect where smb fish would show the regular pattern of paraxial hox gene expression but only later lack dorsal fin mesenchyme formation and consequently eomes expression.

Reviewer 3

Advance summary and potential significance to field

This manuscript addresses an important gap in the literature, the timing and origin of the precursors that give rise to the median fins (dorsal and anal fin), by characterizing a novel dominant phenotype caused by a transgenic insertion. The manuscript is well-written and the conclusions and model are largely well-supported by the data shown.

Comments for the author

Major comments

1. Throughout the manuscript, the authors should incorporate into the legend the number of animals examined and indicate whether the image shown is representative of the animals analyzed. They should do the appropriate quantifications and statistics to support their conclusions. I presume the data is available and point out specific examples of where it should be included in the minor comments section.
2. To support the depletion model shown in Fig 6, the authors should discuss whether the less severe smb+/- mutant phenotype shown in Fig S1 arises because the precursors are “less depleted”. If this is the case, then one would expect that the anal fin defects in such examples would also be less severe and more like wild-type (as long as “expansion” in the two sclerotomal compartments they is affected equally on a fish-by-fish basis). Is this true? Fig S1A should be incorporated into Fig 1; Fig S1B could be easily incorporated as text into the Fig 1 legend. Is the animal shown in Fig. S1 representative? In that fish, it appears that the A/P position of the partial dorsal fin is shifted posteriorly relative to its position in wild-type – this may not support the idea of how the precursors are allocated in an anterior to posterior way proposed by the authors in the discussion. Have the authors looked at the A (alx4a) and P (hand2) transgenes shown in Fig 3 in the smb animals with partial dorsal fins?
3. Fig 5: Very few fish are analyzed (n=6 for 22 hours and n=4 for 24 h), presumably in one experiment.
How carefully was the staging done? For the model shown in Fig. 6, it is important to analyze the dorsal versus ventral sclerotome separately and at additional timepoints that occur after other sclerotomal derivatives differentiate (e.g., between 22 hours and 5.4 SL shown in Fig 2). Don’t the authors expect that expansion, and thus depletion of precursors as differentiation of derivatives occur though time, is more affected in the dorsal versus ventral compartments? In my opinion, this figure is the least convincing in the manuscript, yet crucial for the model proposed. It would be greatly improved with additional timepoints.
4. Fig S7 supports the authors depletion model (and the vertebral defects shown in Fig S7 are incorporated into their model in Fig 6 and thus important for their model). They should consider moving Figure S7 to the main text. Whether or not they move into the main text, they should include n’s in the legend. They should clarify how they are quantifying penetrance – does there just need to be one “aberrant haemal spine” across the entire A/P axis to be counted as affected?
5. In the discussion, the use of the term “sensitivity window” is confusing. The depletion model elicits the idea that sclerotomal cells are multipotent, and that the lack of a dorsal fin in smb fish happens because the other sclerotomal derivatives form first, and thus deplete the precursor population before the dorsal fin forms. Because the authors observe that sclerotomal expansion is affected early, before any derivatives have differentiated (but see point 3), are they implying that median fin precursors are already specified at that stage and especially sensitive? Is

© 2024. Published by The Company of Biologists under the terms of the Creative Commons Attribution License (https://creativecommons.org/licenses/by/4.0/).

that why there are different blue shading (light blue and dark blue) of sclerotomal cells in the model in Fig. 6?

Additional minor comments:
1. Throughout the manuscript, the authors use the term "smb mutant" or "smb fish", and they should clarify whether they mean "smb insertion het" or "smb insertion hom". They should also clarify whether genotype is confirmed via PCR genotyping.
2. In the manuscript, the authors occasionally say "smb functions" or "smb acts" or "is less sensitive to smb". In genetics, wording like that is implying what the wild-type function of a gene is, and in this case is misleading, because there may be no "wild-type" smb gene function. As suggested by the authors, the smb insertion could function to up- or down-regulate a gene that is normally expressed in sclerotome/median fin precursors, but it could also function by activating a gene that is normally never expressed in those cells. The authors should review all such references and change the wording to clarify that they are referring to the dominant phenotype caused by the "smb insertion".
3. Please review all references to figures in the text. There are several errors where the wrong figures and/or panels are referenced. I have tried to indicate these in this section.
4. P. 10, first paragraph: References to Figure 1 are mislabeled, e.g. (Fig. 1B-D') and (Fig. 1D'). There are no
"prime" panels and panel B is a genome picture, not a fish image.
5. Fig. 1 figure, legend, and text: In the figure, diagram (or explain in the legend) which structures are radials versus rays. In panel D, indicate which panels are the dorsal versus anal fin for clarity. How old are the juveniles shown in panel C? In the legend, the description for panel D says that only wild types are shown, but the bottom images are smb mutants. How many animals were stained? Are the images shown representative?
Was an attempt made to quantify the anal fin defects, like what is shown in Fig S1 for the dorsal fin phenotype penetrance?
6. Fig S3 should be moved into the main manuscript.
7. Fig S4: Clarify in the legend why/if there is variable staining of body wall muscle (phallodin) and brighter vertebrae (alizarin red) in the smb fish. The reviewer presumes that this is due to orientation of specimens but if differentiation of sclerotomal derivatives is accelerated in smb mutants, that could modify their model.
For example, if there is continual/gradual expansion of sclerotomal cells (not just between 22-24 hours) in wild-type and smb fish, but accelerated differentiation in smb fish, that could contribute to the depletion of the later-arising derivatives.
8. Fig. 2 figure, legend, and text: The authors state that the phenotypes were observed in the expected Mendelian ratios. They should include the numbers and the chi-square analysis to support the observations.
At the end of this discussion in the text the authors say that "smb functions upstream of fin elongation...".
Because smb is not a gene, and is instead a dominant-acting insertion, they should reword this sentence to acknowledge that fact, like they do in other places in the text. In Fig 2B, the anal fin population does not look reduced to me. Include the n's for panel B in the legend.
9. Fig 3 figure, legend, and text: Include n's in the legend. How many animals were observed and quantified? Were there any smb animals having the rudimentary dorsal fin phenotype and if so, what did alx4 and hand2 expression look like? The last sentence of the text, "This finding suggests a dose-dependent loss of anal fin rays with the addition of smoothback alleles" is awkward. There are only 2 possibilities – het and hom for the insertion.
10. P. 14: cite "data not shown" for the statement, "At 4 dpf, we observe successful labeling of sclerotome-derived cells, including tenocytes, fin mesenchymal cells, and other fibroblasts, consistent with previous lineage analyses (Ma et al., 2018, 2023)."
11. On page 10, citations for Fig S5 are supposed to be for Fig S6. I could not see where the data shown in Fig S5 (about anal fin hand2 radials) were referred to anywhere in the text. The authors can incorporate Fig S5 into Fig 3, or eliminate it altogether.
12. Fig 4: the legend is too brief. Label the dorsal versus anal fin images as in Fig 2B.
13. On page 15, citations for Fig S6 are supposed to be for Fig S7. As mentioned above, the authors should consider moving this figure to the main text.
14. On page 17, in the discussion, the authors say "The caudal fin forms first at 3 dpf and is unaffected by the smoothback insertion." It is strange that it is phrased that way here without

© 2024. Published by The Company of Biologists under the terms of the Creative Commons Attribution License (https://creativecommons.org/licenses/by/4.0/).

mention of the authors data showing that there is only a minor contribution of sclerotome to the caudal fin. Would that be the expectation?
15. Last sentence: Please provide reference(s). “After all, it is changes in regulatory sequences that foster the evolution of developmental mechanisms and major shifts in organismal form”.
16. Concentrations and protocol references of clove oil and tricaine should be included in the methods.

First revision

Author response to reviewers' comments

Reviewer 1 Comments for the Author:
In assessing revision, it would be helpful to comment on a couple of points from the material presented.

In efforts to show position-dependent effect of the *smb* mutant, the authors use repeated insertional analysis to show that the effect was unique to *smb*. However, the authors use the fact that they recover independent lines having similar expression in forming fins – however without a loss of fin growth. This is confusing as the model posed by the authors for the causal effect of the insertion is that it leads to mis or enhanced expression within early progenitor populations. This effect would be thus specific to the insertion site. The finding of similar fin expression in independent lines that are presumed unlinked due to lack of phenotype is confounding, especially as the Sox10 enhancer locus does not normally drive expression in these tissues. Some clarification of this fact/statement would be helpful.
This is a valid point from a reviewer familiar with *sox10* gene regulation. We understand how new insertions also driving reporter gene expression in the fins yet not causing a phenotype might be confounding. Moreover, we agree with the reviewer surmising that the insertion site is critical for the phenotype since the new insertions have wild-type fins. We suspect that the expression of our transgenic construct in fins, even in the new insertions, is likely do to the fact that we cloned a smaller region of the *sox10* regulatory sequences than previous studies. Thus, there may be repressive sequences restricting *sox10* expression from the mesoderm that are included in previous versions but lacking in ours. We now clarify in the results section (lines 190-191 and 198-199) that our construct is different from most of the previously reported *sox10* transgenes and the expression is not fully faithful to endogenous *sox10*, likely owing to the specific construct we use.

The epistasis analysis with longfin highlights classic and really impactful approaches in the use of mutants to discern mechanisms. The longfin analysis places the action of smb prior to growth signals. However, as the hypothesis centers on early populations, it would be useful and more interesting if epistasis analysis can be done on mutants that affect sclerotomal specification to more precisely detail smb action.
An epistasis analysis with a mutant that primarily affects sclerotome specification would provide insight on the developmental mechanism affecting *smb* mutants. Unfortunately, to our knowledge, there are no mutants that affect this specific developmental process yet described. Mutants such as *fss/tbx6* might be a good candidate for this but this mutant does not affect sclerotomal specification and the median fins are fully normal (Fleming et al. 2004). A *pax9* or *nkx3.1* mutant might prove useful in this scenario, but neither of these mutants have yet been reported to have sclerotomal defects. Therefore, we are working on obtaining and creating these mutants to perform this important experiment in future work.

The deduction, or inference of potential neural crest contribution to the caudal fin mesenchyme is not justified/supported. The experiment with Sox10 is not lineage tracing of NCC, but most likely leaky, or pleiotropic expression of Sox10 transgene. The authors would need to do classic lineage analysis, similar to the sclerotome analysis done in this manuscript, to make this connection. The variegation in labeling the authors see is most likely incomplete labeling or silencing of the

© 2024. Published by The Company of Biologists under the terms of the Creative Commons Attribution License (https://creativecommons.org/licenses/by/4.0/).

transgene in mesenchymal clones leading to the formation of the caudal fin anlagen.
The reviewer is correct that we would need a *sox10* lineage tracing experiment to definitively show NCC contribution to the caudal fin. We have removed these assertions from the manuscript to focus solely on the dorsal and anal fin structures affected in the *smb* mutant.

It would also smooth the logic to have F2 dose-dependency analysis moved forward in the results to when the mutant is initially characterized. This will focus the logic of the paper.
We have moved this result section to the initial mutant characterization to smooth the logic of the paper as suggested by the reviewer.

Lastly, while certain parts of the paper were written very well, the impact and integration of these findings were not made clear. This resulted in a disconnected feel of the narrative. What is known of sclerotome contribution, or embryonic lineage of medial fin development overall; why is this important; what does it imply for formation of paired fins and plasticity? Could an evolutionary model be made integrating somitic mesodermal contributions to medial fin formation? In part extending from even from chordates and anamniotes (such as in PMID: 26052418) could be very informative and might lead to experiments to address changes/evolution of somitic mesenchyme contribution
This is an excellent suggestion by the reviewer, we have incorporated this important work in the discussion to highlight our findings and their implications for the evolution of sclerotome and median fins (lines 475-487).

Reviewer 2 Comments for the Author:
Results
smoothback (*smb*) is a dominant mutant with median fin phenotypes caused by a transgenic insertion I encourage revision and publication of this interesting manuscript upon addressing the following points:

p.7 1st paragraph: after screening for GAL4 activity in F1 integrants
The description of the experimental proceedings of screening and identification of the smb mutant is difficult to understand. Please indicate the stage and method you used to identify and screen for GAL4 activity in "stable germline-transmitting integrants" (F1), and which subsequent crossing scheme led to F2 families.
I also don't understand why "the dorsal fin loss is only partial in some families"? Do you refer to individuals within the family showing the smb phenotype?
We now include a thorough description of our initial screening and identification of the *smb* mutant in the results section (lines 144-153). We also now include the frequency of fully absent versus partially absent dorsal fins for four separate families to emphasize the phenotypic variation associated with the *smb* lesion (Fig. 1 and lines 232-235).

Also explicitly state, in the text and also the figure legends, whether you show a heterozygous or homozygous smb mutant (c.f. Figs.1C and S1)
We now explicitly state in the text and figure legends if the individual shown is a heterozygous or homozygous *smb* mutant at every instance.

p.8 1st paragraph: linkage of smb-phenotype and transgenic insertion: here you propose that the transgene "is either causative of the loss of fin phenotype or tightly genetically linked". This implies that the transgene disrupts (or is positioned close to) a genetic regulatory element that is necessary for fin positioning or fin induction.
This is an extremely interesting implication of the mechanism affected in the mutant that you cannot ignore. I suggest, you produce a crispr/CAS deletion of the non-coding region around the insert site of a wild-type chr17:46,219,972 (see next comment) to demonstrate lack of the dorsal

© 2024. Published by The Company of Biologists under the terms of the Creative Commons Attribution License (https://creativecommons.org/licenses/by/4.0/).

fin in a second genetic allele at this site (a deletion allele). This may be a one-generation experiment with sufficiently high gRNA concentration as a proof of principle experiment for the matter of this manuscript.
This is an excellent suggestion. However, the smb insertion locus is very repetitive, and difficult to PCR amplify, let alone design gRNA to specifically mutagenize the region. Nevertheless, per this reviewer's suggestion we designed 6 gRNAs to the best of our ability and injected them. We did not observe any fin loss in these injected mutants, but we cannot be sure we are effectively mutagenizing the locus since PCR amplification was difficult. Therefore, we have not included the negative results from this experiment in this manuscript, yet we will continue to address this important question going forward and hope to include this experiment in future work.

p.8 The smb-causing transgene drives Gal4 in ectopic tissues and does not disrupt a coding sequence. 2nd paragraph: "we crossed a smb mutant to UAS:E2Crimson fish to visualize Gal4 activity": pharyngeal arch labelling: Fig.S2b clearly shows labelling of the hyosymplectic and the ceratohyal, the two elements of the hyoid arch, to a knowledgeable observer. All others may not understand the significance of the image. If you could provide a higher power view of the image, that shows the round structure of the cartilage cells within these red elements, the figure would convince readers that head cartilages are stained.
We have provided a higher power view of the craniofacial cartilage elements in *smb*(*sox10:gal4)* fish including new data colocalizing expression with *sox9:EGFP* to definitively show these are chondrocytes/cartilage in the head (Fig. S2). We also include text to clarify the importance of this result (lines 191-193).

Fig.S2 a&b,c,d: staining in the paraxial mesoderm and mesenchyme of the median fin fold: instead of saying "We conclude that the smb phenotype is genomic integration site-dependent and is not strictly due to Gal4 protein expression in fin mesenchyme. These results motivate the hypothesis that transgene integration into a specific genomic locus affects endogenous gene expression or gene function or both to cause the smb phenotype." You should explicitly state in the text that aside from the labelling of the hyoid arch cartilages, the transgene provides two functions: 1. It can act as an enhancer trap, staining mesodermal cells in the trunk and median fins and 2. at the smb genomic integration site, it mutates a dorsal-fin specific regulatory element which leads to the smb phenotype (hence the above suggestion of creating a second allele by crispr/CAS deletion, to verify this assertion)

This is a good comment and this section requires clarification which we provide here and in the text. We do not believe that the mesodermal labeling in *smb* is strictly due to enhancer trap activity since different (non *smb*) integrations also label these cells. Although we cannot conclude that these new insertions aren't also trapping other mesodermal enhancers, it seems more likely that the shortened *sox10* regulatory sequences in the construct drive mesodermal expression. We clarify in the text that this version of *sox10* regulatory sequences is shorter than those previously reported likely accounting for the mesodermal expression (see above).

p.9/10 The dorsal fin skeleton fails to develop and anal fin skeletal elements are disorganized in smoothback mutants p.10 second paragraph: the sox9a:EGFP-line labels cartilaginous elements, i.e. the endoskeletal radials of the dorsal fin. However, you need to show that, at an earlier stage, the dorsal fin bud/field mesenchyme does not accumulate at its regular position above somites 11 - 15, i.e. if the mesenchyme fails to accumulate, there is no sign or fin induction! You can visualize this by "Nomarsky" optics of the dorsal fin fold of 2 weeks old fish. Bleach pigment if it is in you way or use a pigment mutant so that you can see the initial undifferentiated "bud" mesenchyme. The question is how a lack of fin mesenchyme aggregation comes about. In any case, it is central to the interpretation of the mechanism causing the smb phenotype, viz. axial patterning/fin induction vs. a late failure of fin bud growth/differentiation. It is important that you report evidence that favors one vs. the other cause of the smb phenotype. See next comment

Per this reviewer's request, we now include new DIC/Nomarski images of wild-type and *smb* fish at stages of fin induction. Together, with the *fli1:EGFP* data these results strongly favor the

© 2024. Published by The Company of Biologists under the terms of the Creative Commons Attribution License (https://creativecommons.org/licenses/by/4.0/).

developmental mechanism is due to a lack of fin induction and not growth or differentiation (Fig. 3). Further, in *smb* mutants with a partial dorsal fin (Fig. 1), the cells that do reach the site of future fin development are able to undergo the skeletogenic differentiation program suggesting all the signals for this differentiation are unaffected in *smb* mutants and fin bud induction failure is the mechanism (lines 282-292).

p. 9/10 in comparison to p.6, introduction: in your introduction you introduce the t-box gene eomesa as a necessary factor for dorsal fin formation in zebrafish and carp. Even though this gene was found to be necessary for dorsal fin develoment, its expression pattern has not been reported.
Two other t-box transcription factors, tbx5 and tbx4 are necessary for paired fin/limb formation. They are the earliest markers of pectoral and pelvic fin formation in zebrafish, respectively.
Is eomesa likewise an early marker of the dorsal fin mesenchyme? It would be extremely rewarding if you could undertake this in-situ hybridization, because it would demonstrate the activity of another t-box gene during early fin development and would add to the (ancient) homology argument of unpaired and paired fins the developmental perspective of t-boxes regulating the developmental induction of all appendages!
In addition, you could argue that smb fish don't show early signs of dorsal fin induction instead of arguing smb don't show cartilage radial differentiation in the fin mesenchyme, which is too late.
This is an excellent idea. In fact, we have an active project examining *eomesa* expression and function ongoing in the laboratory. Within this separate study, we performed HCR *in situ* as this reviewer suggests and observed that *eomesa* is indeed expressed in the dorsal and anal fin bud of wild types! These exciting data suggest that *eomesa* may be a t-box median fin gene as this reviewer speculates and have important implications for the (ancient) unpaired and paired fin homology argument. These results are central to the thesis work of another graduate student and we plan to reserve these findings and their implications for a separate study, but share them here for the reviewer to observe (see below). I hope that this same reviewer agrees to review this upcoming manuscript as well!

NOTE: We have removed unpublished data that had been provided for the referees in confidence

p11/12: smb mutants do not establish the dorsal fin bud
p12 first paragraph: "These results motivate the hypothesis that smb affects one of the earliest reported steps in median fin formation":
As pointed out above, you should be able to show that "the earliest steps in median fin formation" is affected in smb mutants and not leave it to a hypothesis, open to discussion.
We have added DIC images of *smb* and wt fish at fin induction stages. We also present data in the manuscript where we show no dorsal fin bud/mesenchyme using a transgenic line (*fli1a:EGFP*) that strongly labels the fin buds in wild types and reveals that they are absent in mutants (Fig. 3).

p.13 The sclerotome is the source of the median fin skeleton this is a strong section in the manuscript! May I encourage you to provide a higher magnification image of Fig.4B such that the reader can actually appreciate the condrocytic distal and proximal radials at the ends of the arrowheads. (You might have to cut away the muscles) There is only little mention in the literature that the endoskeletal radials are of sclerotomal origin, though they were always assumed to be. The neural crest hypothesis also only explicitly addressed the origin of the lepidotichs. Your lineage tracing image could show that both skeletal compartments originate from one progenitor population.
This is a good suggestion. We added zoomed in images of the proximal and distal radial chondrocytes clearly demonstrating Cre mediated recombination in these cells by the *nkx3.1* driver (Fig. S6). We also clarify in the discussion that the sclerotome is the source of both radials and rays (lines 456-458).

p14: Sclerotome expansion is decreased in *smb* mutants please explain what we see in Fig.5 There is a paraxial region of expression lateral to the vertebral column? but there also is a detached "dorsal expression domain". Are these cells the ones that give rise to the dorsal fin? Which pax9 cells did you count in your quantification?
The population of the sclerotome lateral to the vertebral column is known as the "notochord-

© 2024. Published by The Company of Biologists under the terms of the Creative Commons Attribution License (https://creativecommons.org/licenses/by/4.0/).

associated sclerotome” (Ma et al. 2018) and is initially part of the ventral sclerotome region. We now clarify this in the main text (lines 355- 356) and cite appropriate sources. We have not focally mapped the dorsal expression domain and what structures it gives rise to in the larval/adult body. These experiments are active in the lab and we hypothesize that the dorsal domain gives rise to the dorsal fin but we have not yet demonstrated this definitively. However, we have added a section hypothesizing this in the discussion (lines 459-474). For the *pax9* volume analysis, in the original version of the manuscript we quantified the dorsal and ventral domains (which include the notochord-associated cells) together for the 5 somites immediately posterior and anterior to the vent. We now also include separate analyses quantifying the dorsal and ventral domains of the posterior trunk independently at 24 and 26 hpf and find a significant decrease in both at 24 hpf but only in the dorsal but not the ventral domain at 26 hpf (Fig. S7-9). We clearly describe what domains were quantified in each analysis. Thank you for this suggestion that improved the manuscript.

p15/10: Early sclerotome derivatives remain unaffected in the smb mutant
You conclude that "that derivatives of the sclerotome that emerge at early larval stages are unaffected in the smb mutant, while the later forming structures like the median fins are more sensitive".
In this context, it may be interesting to probe for an anterior-posterior axial patterning defect in the smb mutant while mesoderm segmentation per se might be correct. In the paper of Frank van der Hoeven et al. 1995. Mechanisms of Development 54 9-21. the authors point out that the mesodermal axial expression patterns of hoxd genes show the expected collinear expression. Especially hoxd11/hoxd12 show an anterior border of their expression domains at somite 11 and 17, respectively, which marks the region of the anterior margin of dorsal and anal fins. Again, an in situ hybridization with these markers around 22hpf should show if smb shows an a/p patterning defect that would consequently lead to a lack of dorsal fin induction vs. a fin induction defect where smb fish would show the regular pattern of paraxial hox gene expression but only later lack dorsal fin mesenchyme formation and consequently eomes expression.
This is an interesting idea. We performed this experiment as suggested and found that *hoxd* expression was not affected in *smb*. We added these new data to the manuscript (Fig. S5) and discuss that the *smb* insertion likely directly affects fin induction rather than affecting *hox* gene expression which secondarily affects fins (lines 313-326).

***** Reviewer 3 Advance Summary and Potential Significance to Field:
This manuscript addresses an important gap in the literature, the timing and origin of the precursors that give rise to the median fins (dorsal and anal fin), by characterizing a novel dominant phenotype caused by a transgenic insertion. The manuscript is well-written and the conclusions and model are largely well- supported by the data shown.

Reviewer 3 Comments for the Author:
Major comments
1. Throughout the manuscript, the authors should incorporate into the legend the number of animals examined and indicate whether the image shown is representative of the animals analyzed. They should do the appropriate quantifications and statistics to support their conclusions. I presume the data is available and point out specific examples of where it should be included in the minor comments section.

We have added the number of animals examined and p values in the legends throughout. We now include the statistical tests used to analyze the data in the methods (lines 593-597). Thank you for the suggestion.

2. To support the depletion model shown in Fig 6, the authors should discuss whether the less severe smb+/- mutant phenotype shown in Fig S1 arises because the precursors are “less depleted”. If this is the case, then one would expect that the anal fin defects in such examples would also be less severe and more like wild-type (as long as “expansion” in the two sclerotomal compartments they is affected equally on a fish- by-fish basis). Is this true? Fig S1A should be incorporated into Fig 1; Fig S1B could be easily incorporated as text into the Fig 1 legend. Is the animal shown in Fig. S1 representative? In that fish, it

© 2024. Published by The Company of Biologists under the terms of the Creative Commons Attribution License (https://creativecommons.org/licenses/by/4.0/).

appears that the A/P position of the partial dorsal fin is shifted posteriorly relative to its position in wild-type – this may not support the idea of how the precursors are allocated in an anterior to posterior way proposed by the authors in the discussion. Have the authors looked at the A (alx4a) and P (hand2) transgenes shown in Fig 3 in the smb animals with partial dorsal fins?

These are good comments.
1. Regarding partial fin loss individuals being less sclerotome depleted, unfortunately we cannot perform longitudinal studies where we examine the sclerotome and the fin in the same individual which would be required for this experiment. In the discussion, we now speculate that less severe would be less depleted and also detail why we cannot perform these experiments at this time with the available reagents (lines 471-474).
2. Are the anal fins and dorsal fins equally mild in less severe individuals? While the dorsal fin varies in severity, the anal fins tend to be equally affected in all individuals this is quantified by counting fin rays in Fig. S1. Further, per Reviewer 2 above and this reviewers' point below, we now quantify the sclerotome expansion defect separately for the dorsal and ventral compartments finding that the dorsal is more affected at 26 hpf. Again, we cannot examine these fixed samples for their ability to make fins weeks later.
3. We moved the supplemental figure to the main Fig 1 per this reviewer's request. Further, we now include the frequency of these partials in several different families in the main figure. The dorsal fin is not shifted posteriorly in this individual. Counting vertebrae indicates that the anterior portion of the wild type and the partial fin are at the same A-P position. Per this reviewer's suggestion we include new data looking at the posterior marker *hand2* in partial dorsal fins. We find that the number of posterior rays indicated by *hand2* expression is reduced in partial fin loss individuals (Fig. S4, described in lines 307-309). We did the *alx4a* transgene experiment as suggested.
Unfortunately, we were unable to recover any partial fin loss individuals from families expressing the *alx4a* transgene in time for this revision.

3. Fig 5: Very few fish are analyzed (n=6 for 22 hours and n=4 for 24 h), presumably in one experiment. How carefully was the staging done? For the model shown in Fig. 6, it is important to analyze the dorsal versus ventral sclerotome separately and at additional timepoints that occur after other sclerotomal derivatives differentiate (e.g., between 22 hours and 5.4 SL shown in Fig 2). Don't the authors expect that expansion, and thus depletion of precursors as differentiation of derivatives occur though time, is more affected in the dorsal versus ventral compartments? In my opinion, this figure is the least convincing in the manuscript, yet crucial for the model proposed. It would be greatly improved with additional timepoints.

We agree that additional timepoints would greatly improve this figure and now include them in the paper (Fig. S7-9). Notably, we include a later stage after other sclerotomal derivatives start to differentiate. We also now quantify dorsal and ventral sclerotomes separately as described above. The staging was done very carefully according to Kimmel et al 1996.

4. Fig S7 supports the authors depletion model (and the vertebral defects shown in Fig S7 are incorporated into their model in Fig 6 and thus important for their model). They should consider moving Figure S7 to the main text. Whether or not they move into the main text, they should include n's in the legend. They should clarify how they are quantifying penetrance - does there just need to be one "aberrant haemal spine" across the entire A/P axis to be counted as affected?

We moved these findings to the main text per this reviewer's suggestion. We now clarify how the phenotype was quantified in the text (lines 378-380) and include n's in the legend in addition to the figure.

© 2024. Published by The Company of Biologists under the terms of the Creative Commons Attribution License (https://creativecommons.org/licenses/by/4.0/).

5. In the discussion, the use of the term “sensitivity window” is confusing. The depletion model elicits the idea that sclerotomal cells are multipotent, and that the lack of a dorsal fin in smb fish happens because the other sclerotomal derivatives form first, and thus deplete the precursor population before the dorsal fin forms. Because the authors observe that sclerotomal expansion is affected early, before any derivatives have differentiated (but see point 3), are they implying that median fin precursors are already specified at that stage and especially sensitive? Is that why there are different blue shading (light blue and dark blue) of sclerotomal cells in the model in Fig. 6?

We have corrected to all same shade of blue in the model figure. We have removed all mention of the sensitivity window from the discussion to strengthen arguments for our depletion model. We do not yet know if median fin precursors are already specified at that stage, this is an active avenue of investigation in the lab.

Additional minor comments:
1. Throughout the manuscript, the authors use the term “smb mutant” or “smb fish”, and they should clarify whether they mean “smb insertion het” or “smb insertion hom”. They should also clarify whether genotype is confirmed via PCR genotyping.

We now explicitly state whenever we refer to the heterozygous or homozygous condition in each case. All genotypes were confirmed via PCR genotyping or Gal4 activity which we now clearly state.

2. In the manuscript, the authors occasionally say “smb functions” or “smb acts” or “is less sensitive to smb”. In genetics, wording like that is implying what the wild-type function of a gene is, and in this case is misleading, because there may be no “wild-type” smb gene function. As suggested by the authors, the smb insertion could function to up- or down-regulate a gene that is normally expressed in sclerotome/median fin precursors, but it could also function by activating a gene that is normally never expressed in those cells. The authors should review all such references and change the wording to clarify that they are referring to the dominant phenotype caused by the “smb insertion”.

We changed the text accordingly throughout as this reviewer suggests.

3. Please review all references to figures in the text. There are several errors where the wrong figures and/or panels are referenced. I have tried to indicate these in this section.

We have carefully revised all the references to figures in the text and ensured the proper panels are referenced. We apologize for the confusion and thank you for pointing this out.

4. P. 10, first paragraph: References to Figure 1 are mislabeled, e.g. (Fig. 1B-D’) and (Fig. 1D’). There are no “prime” panels and panel B is a genome picture, not a fish image.

We have corrected this.

5. Fig. 1 figure, legend, and text: In the figure, diagram (or explain in the legend) which structures are radials versus rays. In panel D, indicate which panels are the dorsal versus anal fin for clarity.

© 2024. Published by The Company of Biologists under the terms of the Creative Commons Attribution License (https://creativecommons.org/licenses/by/4.0/).

How old are the juveniles shown in panel C? In the legend, the description for panel D says that only wild types are shown, but the bottom images are smb mutants. How many animals were stained? Are the images shown representative? Was an attempt made to quantify the anal fin defects, like what is shown in Fig S1 for the dorsal fin phenotype penetrance?

We have labeled radials vs rays in the figure and added the age of juveniles in the legend. Penetrance of anal fin A-P reductions is 100% which we indicate in the text (lines 153).

6. Fig S3 should be moved into the main manuscript.

We moved this figure to the main text.

7. Fig S4: Clarify in the legend why/if there is variable staining of body wall muscle (phallodin) and brighter vertebrae (alizarin red) in the smb fish. The reviewer presumes that this is due to orientation of specimens, but if differentiation of sclerotomal derivatives is accelerated in smb mutants, that could modify their model. For example, if there is continual/gradual expansion of sclerotomal cells (not just between 22-24 hours) in wild-type and smb fish, but accelerated differentiation in smb fish, that could contribute to the depletion of the later-arising derivatives.

We state that the variable staining is due to specimen orientation in the figure legend for clarification (Figure S3).

8. Fig. 2 figure, legend, and text: The authors state that the phenotypes were observed in the expected Mendelian ratios. They should include the numbers and the chi-square analysis to support the observations. At the end of this discussion in the text the authors say that "smb functions upstream of fin elongation…". Because smb is not a gene, and is instead a dominant-acting insertion, they should reword this sentence to acknowledge that fact, like they do in other places in the text. In Fig 2B, the anal fin population does not look reduced to me. Include the n's for panel B in the legend.

We made all of these changes as suggested.

9. Fig 3 figure, legend, and text: Include n's in the legend. How many animals were observed and quantified? Were there any smb animals having the rudimentary dorsal fin phenotype and if so, what did alx4 and hand2 expression look like? The last sentence of the text, "This finding suggests a dose-dependent loss of anal fin rays with the addition of smoothback alleles" is awkward. There are only 2 possibilities – het and hom for the insertion.

We examined *hand2:EGFP* expression in the partial dorsal fins of *smb* mutants and find a similar trend as in the anal fins, see in-depth response above. We added these analyses to the manuscript and corrected the wording of the "dose-dependent" finding as suggested.

10. P. 14: cite "data not shown" for the statement, "At 4 dpf, we observe successful labeling of sclerotome- derived cells, including tenocytes, fin mesenchymal cells, and other fibroblasts, consistent with previous lineage analyses (Ma et al., 2018, 2023)."

We added "data not shown" to this statement.

© 2024. Published by The Company of Biologists under the terms of the Creative Commons Attribution License (https://creativecommons.org/licenses/by/4.0/).

11. On page 10, citations for Fig S5 are supposed to be for Fig S6. I could not see where the data shown in Fig S5 (about anal fin hand2 radials) were referred to anywhere in the text. The authors can incorporate Fig S5 into Fig 3, or eliminate it altogether.

We eliminated this figure and added its data and statistics to the main text.

12. Fig 4: the legend is too brief. Label the dorsal versus anal fin images as in Fig 2B.

We labeled the dorsal and anal fin in the figures as in 2B. We now include the n for this experiment.

13. On page 15, citations for Fig S6 are supposed to be for Fig S7. As mentioned above, the authors should consider moving this figure to the main text.

This figure has now been moved to the main text.

14. On page 17, in the discussion, the authors say “The caudal fin forms first at 3 dpf and is unaffected by the smoothback insertion.” It is strange that it is phrased that way here without mention of the authors data showing that there is only a minor contribution of sclerotome to the caudal fin. Would that be the expectation?

Per Reviewer 1’s request, we removed all mention of the caudal fin origin and now focus only on the dorsal and anal fins, skeletal structures affected in the *smb* mutant.

15. Last sentence: Please provide reference(s). “After all, it is changes in regulatory sequences that foster the evolution of developmental mechanisms and major shifts in organismal form”.

We added references for this statement.

16. Concentrations and protocol references of clove oil and tricaine should be included in the methods.

We now include the concentrations used in the methods section.

<u>Second decision letter</u>

MS ID#: DEVELOP/2024/203025

MS TITLE: The sclerotome is the source of the dorsal and anal fin skeleton and its expansion is required for median fin development

AUTHORS: Raisa Bailon-Zambrano, Margaret Keating, Emily C. Sales, Abigail R. Nichols, Grace E. Gustafson, Colette A Hopkins, Katrinka M. Kocha, Peng Huang, Lindsey Barske, and James T. Nichols

© 2024. Published by The Company of Biologists under the terms of the Creative Commons Attribution License (https://creativecommons.org/licenses/by/4.0/).

I have now received all the referees reports on the above manuscript, and have reached a decision. The referees' comments are appended below, or you can access them online: please go to BenchPress and click on the 'Manuscripts with Decisions' queue in the Author Area.

The overall evaluation is positive and we would like to publish a revised manuscript in Development, provided that the referees' remaining comments can be satisfactorily addressed. Please attend to all of the reviewers' comments in your revised manuscript and detail them in your point-by-point response. If you do not agree with any of their criticisms or suggestions explain clearly why this is so. If it would be helpful, you are welcome to contact us to discuss your revision in greater detail. Please send us a point-by-point response indicating your plans for addressing the referees' comments, and we will look over this and provide further guidance.

Reviewer 1

Advance summary and potential significance to field

This is an analysis of a revision. Please see previous summary statemetn.

Comments for the author

I think the authors have done a commendable job is sufficiently addressing all questions put forward in previous review. The paper is greatly improved and is in great shape for acceptance. Further, I fully agree with the excitement of parallels with the tbx in sclerotome and medial/paired fins as raised by reviewer 2 - but agree this would be too much for this paper.

One small change would help clarify a sentence in the results "we reasoned that gain of fin phenotypes found in lof might genetically interact with the loss of fin.." Longfin is not really a gain of fin - it is a gain of function, or increased fin growth mutant - the latter being probably the best for this comparison. Also it would be good to cite Iovine and Johnson and/or zf screen paper 1996 instead of Stewart 2021 or even Daane 2021, as this is the initial lof description. Daane 2021 and Stewart 2021 detail the molecular causes and mathematical allometry, respectively.

I have no further suggestions to a fine paper.

Reviewer 2

Advance summary and potential significance to field

- the paper describes a transgenic insertion mutant providing an inroad into zebrafish median fin development at late larval developmental stages that are understudied.
- the study identifies the sclerotome as source tissue for endo- and expsceletal elements of the median fins

Comments for the author

The following text includes the comments of the first review, Your response and my current reply to your response.
As you will see, in many cases I agree with your response to my initial comment, in some I suggest changes in wording or caution with the interpretation.

There is one case of concern. This relates to your analysis of precursor expansion during early embryonic stages. While you find reduction of pax9 expressing sclerotome tissue at 24hpf and 26 hpf you fail to show this at 30hpf, though your Fig S9 appears to show otherwise. I suggest you revisit your quantification or apply a more robust method of quantification e.g. EdU, see second to last reply below.

Point by point response to the Author:

© 2024. Published by The Company of Biologists under the terms of the Creative Commons Attribution License (https://creativecommons.org/licenses/by/4.0/).

p.7 1st paragraph: after screening for GAL4 activity in F1 integrants The description of the experimental proceedings of screening and identification of the smb mutant is difficult to understand. Please indicate the stage and method you used to identify and screen for GAL4 activity in "stable germline-transmitting integrants" (F1), and which subsequent crossing scheme led to F2 families.
I also don't understand why "the dorsal fin loss is only partial in some families"? Do you refer to individuals within the family showing the smb phenotype?
Your response: We now include a thorough description of our initial screening and identification of the smb mutant in the results section (lines 144-153). We also now include the frequency of fully absent versus partially absent dorsal fins for four separate families to emphasize the phenotypic variation associated with the smb lesion (Fig. 1 and lines 232-235).
Reviewer2 reply: valid answer Also explicitly state, in the text and also the figure legends, whether you show a heterozygous or homozygous smb mutant (c.f. Figs.1C and S1)
Your response: We now explicitly state in the text and figure legends if the individual shown is a heterozygous or homozygous smb mutant at every instance.
Reviewer2 reply: many done except Figs.3C, 6,A,B,D, S5, S7, S8, S9 p.8 1st paragraph: linkage of smb-phenotype and transgenic insertion: here you propose that the transgene "is either causative of the loss of fin phenotype or tightly genetically linked". This implies that the transgene disrupts (or is positioned close to) a genetic regulatory element that is necessary for fin positioning or fin induction.
This is an extremely interesting implication of the mechanism affected in the mutant that you cannot ignore. I suggest, you produce a crispr/CAS deletion of the non-coding region around the insert site of a wild-type chr17:46,219,972 (see next comment) to demonstrate lack of the dorsal fin in a second genetic allele at this site (a deletion allele). This may be a one-generation experiment with sufficiently high gRNA concentration as a proof of principle experiment for the matter of this manuscript.
Your response: This is an ¬excellent suggestion. However, the smb insertion locus is very repetitive, and difficult to PCR amplify, let alone design gRNA to specifically mutagenize the region. Nevertheless, per this reviewer’s suggestion we designed 6 gRNAs to the best of our ability and injected them. We did not observe any fin loss in these injected mutants, but we cannot be sure we are effectively mutagenizing the locus since PCR amplification was difficult. Therefore, we have not included the negative results from this experiment in this manuscript, yet we will continue to address this important question going forward and hope to include this experiment in future work.
Reviewer2 reply: Unfortunate, the suggested experiment doesn’t seem to be within the scope of a revision.
p.8 The smb-causing transgene drives Gal4 in ectopic tissues and does not disrupt a coding sequence. 2nd paragraph: "we crossed a smb mutant to UAS:E2Crimson fish to visualize Gal4 activity": pharyngeal arch labelling: Fig.S2b clearly shows labelling of the hyosymplectic and the ceratohyal, the two elements of the hyoid arch, to a knowledgeable observer. All others may not understand the significance of the image. If you could provide a higher power view of the image, that shows the round structure of the cartilage cells within these red elements, the figure would convince readers that head cartilages are stained.
Your response: We have provided a higher power view of the craniofacial cartilage elements in smb(sox10:gal4) fish including new data colocalizing expression with sox9:EGFP to definitively show these are chondrocytes/cartilage in the head (Fig. S2). We also include text to clarify the importance of this result (lines 191-193).
Reviewer2 reply: The supplementary Fig. 2B is beautiful and clear. Please include in your figure legend and in the text (line192) that all elements of the first arch are visible: Meckel’s cartilage, hyosymplectic and ceratohyal and don’t leave out one or the other in your figure legend and text. Please don’t show Fig.S2C. The notochord staining of the sox9aEGFP transgenic line is not helpful and appears to be a peculiarity of this line. You also don’t even mention it in the text nor in the figure legend. Please instead include Fig.S2 B form version1 (embryo tail only!) because it shows neural tube staining and fin mesenchymal staining much better than the new Fig.S2C.I’m sorry that I have to confess that I don’t understand which tissue is stained in Fig. S2D. I’d expect to see somites/myotomes at 5dpf in the paraxial mesoderm but the tissue is not segmented. Is this non-migrated sclerotome? Please add an explanation to the legend of S2.
Figure legend S2C: “Caret indicates ectopic labeling of caudal fin fold fibroblasts by sox10:Gal4;UAS:E2Crimson.
Cancel “ectopic”! You just document the expression of your transgenic line as it is. The mesoderm expression is the topic of the paper anyway.

© 2024. Published by The Company of Biologists under the terms of the Creative Commons Attribution License (https://creativecommons.org/licenses/by/4.0/).

Fig.S2 a&b,c,d: staining in the paraxial mesoderm and mesenchyme of the median fin fold: instead of saying "We conclude that the smb phenotype is genomic integration site-dependent and is not strictly due to Gal4 protein expression in fin mesenchyme. These results motivate the hypothesis that transgene integration into a specific genomic locus affects endogenous gene expression or gene function or both to cause the smb phenotype." You should explicitly state in the text that aside from the labelling of the hyoid arch cartilages, the transgene provides two functions: 1. It can act as an enhancer trap, staining mesodermal cells in the trunk and median fins and 2. at the smb genomic integration site, it mutates a dorsal-fin specific regulatory element which leads to the smb phenotype (hence the above suggestion of creating a second allele by crispr/CAS deletion, to verify this assertion)
Your response: This is a good comment and this section requires clarification which we provide here and in the text. We do not believe that the mesodermal labeling in smb is strictly due to enhancer trap activity since different (non smb) integrations also label these cells. Although we cannot conclude that these new insertions aren't also trapping other mesodermal enhancers, it seems more likely that the shortened sox10 regulatory sequences in the construct drive mesodermal expression. We clarify in the text that this version of sox10 regulatory sequences is shorter than those previously reported likely accounting for the mesodermal expression (see above).
Reviewer2 reply: I agree with your interpretation. Your argument of 3 independent transgenic lines showing the same mesodermal expression is convincing. Line 208 replace "presumably" with "likely". Line 212 replace "other" with "in our".
Lines 215-216 proposal: These results motivate the hypothesis that transgene integration specifically into the smb genomic locus …

p.9/10 The dorsal fin skeleton fails to develop and anal fin skeletal elements are disorganized in smoothback mutants p.10 second paragraph: the sox9a:EGFP-line labels cartilaginous elements, i.e. the endoskeletal radials of the dorsal fin. However, you need to show that, at an earlier stage, the dorsal fin bud/field mesenchyme does not accumulate at its regular position above somites 11 – 15, i.e. if the mesenchyme fails to accumulate, there is no sign or fin induction! You can visualize this by "Nomarsky" optics of the dorsal fin fold of 2 weeks old fish. Bleach pigment if it is in you way or use a pigment mutant so that you can see the initial undifferentiated "bud" mesenchyme. The question is how a lack of fin mesenchyme aggregation comes about. In any case, it is central to the interpretation of the mechanism causing the smb phenotype, viz. axial patterning/fin induction vs. a late failure of fin bud growth/differentiation. It is important that you report evidence that favors one vs. the other cause of the smb phenotype. See next comment Your response: Per this reviewer's request, we now include new DIC/Nomarski images of wild-type and smb fish at stages of fin induction. Together, with the fli1:EGFP data these results strongly favor the developmental mechanism is due to a lack of fin induction and not growth or differentiation (Fig. 3). Further, in smb mutants with a partial dorsal fin (Fig. 1), the cells that do reach the site of future fin development are able to undergo the skeletogenic differentiation program suggesting all the signals for this differentiation are unaffected in smb mutants and fin bud induction failure is the mechanism (lines 282-292).
Reviewer2 reply: Fig.3 is convincing. The lack of mesenchymal accumulation is shown well, also in the fli1:EGFP transgenes.
Text: line 283: replace "look at" by "view of". Line 285: replace "ectomesenchyme" by "mesenchymal": It's true that fli1:EGFP labes ectomesenchymal i.e. crest derived cartilage progenitors. But this is not what you wish to imply wih the median fins! You show by lineage labelling the fin mesenchymal cells are sclerotome-derived. Who knows why the fli-line also labels fin mesenchyme? Just avoid the unnecessary theory-laden "ecto"mesenchyme.
Line 291: the earliest reported step in median fin formation, potentially induction of fin bud mesenchymal progenitors…..
The sclerotomal progenitors might be induced but fail to migrate, hence I suggest to downscale your hypothesis Line303: hand2:EGPF labels the posteriormost rays … give a reference

p. 9/10 in comparison to p.6, introduction: in your introduction you introduce the t-box gene eomesa as a necessary factor for dorsal fin formation in zebrafish and carp. Even though this gene was found to be necessary for dorsal fin develoment, its expression pattern has not been reported. Two other t-box transcription factors, tbx5 and tbx4 are necessary for paired fin/limb formation. They are the earliest markers of pectoral and pelvic fin formation in zebrafish, respectively.

© 2024. Published by The Company of Biologists under the terms of the Creative Commons Attribution License (https://creativecommons.org/licenses/by/4.0/).

Is eomesa likewise an early marker of the dorsal fin mesenchyme? It would be extremely rewarding if you could undertake this in-situ hybridization, because it would demonstrate the activity of another t-box gene during early fin development and would add to the (ancient) homology argument of unpaired and paired fins the developmental perspective of t-boxes regulating the developmental induction of all appendages!
In addition, you could argue that smb fish don't show early signs of dorsal fin induction instead of arguing smb don't show cartilage radial differentiation in the fin mesenchyme, which is too late.
Your response: This is an excellent idea. In fact, we have an active project examining eomesa expression and function ongoing in the laboratory. Within this separate study, we performed HCR in situ as this reviewer suggests and observed that eomesa is indeed expressed in the dorsal and anal fin bud of wild types! These exciting data suggest that eomesa may be a t-box median fin gene as this reviewer speculates and have important implications for the (ancient) unpaired and paired fin homology argument. These results are central to the thesis work of another graduate student and we plan to reserve these findings and their implications for a separate study, but share them here for the reviewer to observe (see below). I hope that this same reviewer agrees to review this upcoming manuscript as well!

p11/12: smb mutants do not establish the dorsal fin bud p12 first paragraph: "These results motivate the hypothesis that smb affects one of the earliest reported steps in median fin formation":
As pointed out above, you should be able to show that "the earliest steps in median fin formation" is affected in smb mutants and not leave it to a hypothesis, open to discussion.
We have added DIC images of smb and wt fish at fin induction stages. We also present data in the manuscript where we show no dorsal fin bud/mesenchyme using a transgenic line (fli1a:EGFP) that strongly labels the fin buds in wild types and reveals that they are absent in mutants (Fig. 3).
Reviewer2 reply: Thank you for sharing the interesting eomesa in-situs. The hypothesis that “smb disrupts the earliest reported steps in median fin formation” is supported by the demonstration of lack of the mesenchymal anlagen.

p.13 The sclerotome is the source of the median fin skeleton this is a strong section in the manuscript! May I encourage you to provide a higher magnification image of Fig.4B such that the reader can actually appreciate the condrocytic distal and proximal radials at the ends of the arrowheads. (You might have to cut away the muscles) There is only little mention in the literature that the endoskeletal radials are of sclerotomal origin, though they were always assumed to be. The neural crest hypothesis also only explicitly addressed the origin of the lepidotichs. Your lineage tracing image could show that both skeletal compartments originate from one progenitor population.
Your response: This is a good suggestion. We added zoomed in images of the proximal and distal radial chondrocytes clearly demonstrating Cre mediated recombination in these cells by the nkx3.1 driver (Fig. S6). We also clarify in the discussion that the sclerotome is the source of both radials and rays (lines 456-458).
Reviewer2 reply: Good and clear images and good conclusions

p14: Sclerotome expansion is decreased in smb mutants please explain what we see in Fig.5 There is a paraxial region of expression lateral to the vertebral column? but there also is a detached "dorsal expression domain". Are these cells the ones that give rise to the dorsal fin? Which pax9 cells did you count in your quantification?
Your response: The population of the sclerotome lateral to the vertebral column is known as the “notochord-associated sclerotome” (Ma et al. 2018) and is initially part of the ventral sclerotome region. We now clarify this in the main text (lines 355- 356) and cite appropriate sources. We have not focally mapped the dorsal expression domain and what structures it gives rise to in the larval/adult body. These experiments are active in the lab and we hypothesize that the dorsal domain gives rise to the dorsal fin but we have not yet demonstrated this definitively. However, we have added a section hypothesizing this in the discussion (lines 459-474). For the pax9 volume analysis, in the original version of the manuscript we quantified the dorsal and ventral domains (which include the notochord-associated cells) together for the 5 somites immediately posterior and anterior to the vent. We now also include separate analyses quantifying the dorsal and ventral domains of the posterior trunk independently at 24 and 26 hpf and find a significant decrease in both at 24 hpf but only in the dorsal but not the ventral domain at 26 hpf (Fig. S7-9). We clearly

© 2024. Published by The Company of Biologists under the terms of the Creative Commons Attribution License (https://creativecommons.org/licenses/by/4.0/).

describe what domains were quantified in each analysis. Thank you for this suggestion that improved the manuscript.
Reviewer2 reply: Thank you for more precisely describing the pax9 domains and for the quantification

Your quantification of 24hpf and 26hpf pax9 domains give support to your depletion model (line 424 ff.) if we assume that the measurable depletion between 24-26 hpf leads to a smaller precursor population that cannot be compensated for at later stages. The idea is valid, however, you argue yourself that by 30hpf, the reduction of the sclerotome is not measurable any more.

Fig.S9: the smb image exhiits a differently sized pax9 domain than the wt at 30hpf, which I would have expected according to your analysis at 24 and 26hpf. I realize, there are expressivity differences between individual embryos but this one doesn't show that wt and smb have identical amounts of pax9 expressing precursors. The smb embryo you show has clearly weaker pax9 expression than the wt, does it even have segmentation problems (?) as it doesn't show the chevron shapes of the wt-somites. It is difficult to acknowledge that the pax9 domains are alike in smb and wt at 30hpf. Are they really?

If so, this is a problem, because the depletion-model relies on the lack of precursors. Have you tried to use a different measure to quantify your precursor pool, e.g. EdU? It would be easier to my mind to accept an early embryonic depletion that leads to lack of material at later larval developmental stages thus affecting the unpaired fins rather than observing an only transient retardation of precursor development that is compensated for only 4 hours later by 30hpf.

Perhaps I am missing an argument here but I suggest you revisit the 30hpf results and perhaps apply a more robust method of quantification.

p15/10: Early sclerotome derivatives remain unaffected in the smb mutant You conclude that "that derivatives of the sclerotome that emerge at early larval stages are unaffected in the smb mutant, while the later forming structures like the median fins are more sensitive".
In this context, it may be interesting to probe for an anterior-posterior axial patterning defect in the smb mutant while mesoderm segmentation per se might be correct. In the paper of Frank van der Hoeven et al. 1995. Mechanisms of Development 54 9-21. the authors point out that the mesodermal axial expression patterns of hoxd genes show the expected collinear expression. Especially hoxd11/hoxd12 show an anterior border of their expression domains at somite 11 and 17, respectively, which marks the region of the anterior margin of dorsal and anal fins. Again, an in situ hybridization with these markers around 22hpf should show if smb shows an a/p patterning defect that would consequently lead to a lack of dorsal fin induction vs. a fin induction defect where smb fish would show the regular pattern of paraxial hox gene expression but only later lack dorsal fin mesenchyme formation and consequently eomes expression.
Your response: This is an interesting idea. We performed this experiment as suggested and found that hoxd expression was not affected in smb. We added these new data to the manuscript (Fig. S5) and discuss that the smb insertion likely directly affects fin induction rather than affecting hox gene expression which secondarily affects fins (lines 313-326).
Reviewer2 reply: Line 323-326: Rephrase your conclusion from the hoxd12 expression pattern or maybe put it into a different context, see last paragraph. Here the lengthy reasoning:

Even though "fin induction" is a pre-molecular term describing a process that leads to aggregation of the fin mesenchyme as the earliest visible read out, this process is generally thought to include a "positioning" component. This "positioning" has been proposed to be dependent on the borders between axial "segments", that can be visualized by the collinear expression of hox genes. Thus, if you find that the expression pattern of a hox gene is not disturbed in smb mutants, this actually argues in favor of a normal patterning process and consequently not in favor of disruption of limb induction due to confounded identities of the relevant somites. The "induction" defect that leads to lack of a mesenchymal aggregation must thus develop from a defect other than axial pattern identity issues.

You have shown the lack of a mesenchymal condensation in smb-transgenics. Thus, there is a process blocked at an early stage of fin development. Showing correct axial expression of hoxd12 alone may show that axial patterning is not disrupted and therefore this component of "fin

© 2024. Published by The Company of Biologists under the terms of the Creative Commons Attribution License (https://creativecommons.org/licenses/by/4.0/).

induction" in not affected (though expression of a single hox gene is perhaps not sufficient for such a conclusion.)
Your observation is ,however, in favor of your hypothesis that the lack of dorsal fin mesenchyme is a problem of "sclerotme expansion" thus strengthening your view, that smb controls precursor supply. You may more easily discuss your hoxd12 results in this context.

Reviewer 3

Advance summary and potential significance to field

This reviewer was enthusiastic about the manuscript after first review and finds the manuscript additionally improved after revision. The manuscript addresses an important gap in the literature, the timing and origin of the precursors that give rise to the median fins (dorsal and anal fin), by characterizing a novel dominant phenotype caused by a transgenic insertion. The manuscript is well-written and the conclusions and model are well-supported by the data shown.

Comments for the author

The reviewer feels that that the authors did not fully address minor comments 1 and 2 from the previous review regarding nomenclature on "smb zygosity" and "smb function". We found several instances where the terminology is still confusing and think that the authors are confounding their message. Although somewhat minor, these are easy fixes that should be addressed before publication.
The authors should read the ZFIN Nomenclature Conventions (https://zfin.atlassian.net/wiki/spaces/general/pages/1818394635/ZFIN+Zebrafish+Nomenclature+Convention s), Section 4.3 on Transgenic lines and constructs, and specifically 4.3.3 on Transgenic lines, where it states
"Transgenic lines are of two types, those that are known to create alleles of genes and those that are not known to create alleles of genes". Authors should use the appropriate convention for the latter. Using these guidelines, they should assign an allele designation for the insertion allele and clearly define this designation in the Methods section (they cite allele designations for all other lines they use in the paper in the methods but not their own). They do assign an allele designation on Line 143, but it is not consistent with ZFIN nomenclature guidelines. Since they only have one smb insertion allele, it's fine to state the zygosity (and not the specific allele designation) every time, once it has been initially defined.

Examples of where terminology is confusing in the text (may not include all examples; authors are encouraged to carefully review for other instances in the text, figures, and legends):
- Line 127: clarify by saying "dominant mutant"
- Lines 132, 136, 186, 274, 294, 297, 300, 302, 304, 307, 358, 362, 368, 369, 406, 420 : zygosity is not stated
- Line 137: "depletion in smb mutants"
- Line 169: "to determine whether homozygosity for the smb transgene" – if there allele were defined in the section above, this would streamline and clarify the description here, e.g. "to determine whether homozygosity for the smb transgenic insertion allele, smbco3021Tg, …"
- Line 187: clarify what "double transgenic fish" means. Here you are "using" smb for the transgene that caused the insertion allele, correct? This may be confusing to a reader.
- Line 263: "two dominant mutants". Mutations, alleles, and phenotypes are dominant. Just stating the genotypes of the parents would be clearer.
- Line 266-8: In this section (line 266-268, it would be easier if the authors just clearly and consistently stated the genotypes of the phenotypic classes. They do state the lof zygosity, but not smb zygosity, in the Figure associated with this text.
- Line 275: "smb defect" is unclear
- Line 289: "stage-matched smb", zyogosity and mutant not stated
- Line 290: "smb disrupts" implies there is a wild-type smb that functions to disrupt a process
- Line 309: term "partial mutants" is unclear
- Line 411: "less sensitive to smb" – the meaning here is unclear.

© 2024. Published by The Company of Biologists under the terms of the Creative Commons Attribution License (https://creativecommons.org/licenses/by/4.0/).

Second revision

Author response to reviewers' comments

Reviewer 1 Advance Summary and Potential Significance to Field:
This is an analysis of a revision. Please see previous summary statement.

Reviewer 1 Comments for the Author:
I think the authors have done a commendable job is sufficiently addressing all questions put forward in previous review. The paper is greatly improved and is in great shape for acceptance. Further, I fully agree with the excitement of parallels with the tbx in sclerotome and medial/paired fins as raised by reviewer 2 - but agree this would be too much for this paper.
One small change would help clarify a sentence in the results "we reasoned that gain of fin phenotypes found in lof might genetically interact with the loss of fin.." Longfin is not really a gain of fin - it is a gain of function, or increased fin growth mutant - the latter being probably the best for this comparison. Also it would be good to cite Iovine and Johnson and/or zf screen paper 1996 instead of Stewart 2021 or even Daane 2021, as this is the initial lof description. Daane 2021 and Stewart 2021 detail the molecular causes and mathematical allometry, respectively.

We would like to thank Reviewer 1 for their efforts on the original and the revised manuscript. We agree with this textual suggestion and made the change as recommended. We also included the Iovine and van Eeden citations in addition to Daane and Stewart. We thank the reviewer for carefully reading our manuscript and their thoughtful comments.
I have no further suggestions to a fine paper.

***** Reviewer 2 Advance Summary and Potential Significance to Field:
- the paper describes a transgenic insertion mutant providing an inroad into zebrafish median fin development at late larval developmental stages that are understudied.
- the study identifies the sclerotome as source tissue for endo- and expsceletal elements of the median fins

Reviewer 2 Comments for the Author:
The following text includes the comments of the first review, Your response and my current reply to your response.
As you will see, in many cases I agree with your response to my initial comment, in some I suggest changes in wording or caution with the interpretation.
There is one case of concern. This relates to your analysis of precursor expansion during early embryonic stages. While you find reduction of pax9 expressing sclerotome tissue at 24hpf and 26 hpf you fail to show this at 30hpf, though your Fig S9 appears to show otherwise. I suggest you revisit your quantification or apply a more robust method of quantification e.g. EdU, see second to last reply below.

We thank Reviewer 2 for reviewing our manuscript, see below for point-by-point response.

Point by point response to the Author:
p.7 1st paragraph: after screening for GAL4 activity in F1 integrants
The description of the experimental proceedings of screening and identification of the smb mutant is difficult to understand. Please indicate the stage and method you used to identify and screen for GAL4 activity in "stable germline-transmitting integrants" (F1), and which subsequent crossing scheme led to F2 families.
I also don't understand why "the dorsal fin loss is only partial in some families"? Do you refer to individuals within the family showing the smb phenotype?
Your response: We now include a thorough description of our initial screening and identification of the smb mutant in the results section (lines 144-153). We also now include the frequency of fully absent versus partially absent dorsal fins for four separate families to emphasize the phenotypic variation associated with the smb lesion (Fig. 1 and lines 232-235).
Reviewer2 reply: valid answer

© 2024. Published by The Company of Biologists under the terms of the Creative Commons Attribution License (https://creativecommons.org/licenses/by/4.0/).

We thank the reviewer for this helpful comment which strengthens our manuscript and clarifies our findings.

Also explicitly state, in the text and also the figure legends, whether you show a heterozygous or homozygous smb mutant (c.f. Figs.1C and S1)
Your response: We now explicitly state in the text and figure legends if the individual shown is a heterozygous or homozygous smb mutant at every instance.
Reviewer2 reply: many done except Figs.3C, 6,A,B,D, S5, S7, S8, S9
We have carefully added the zygosity in all these cases. We apologize for any confusion and thank you for your attention to detail.

p.8 1st paragraph: linkage of smb-phenotype and transgenic insertion: here you propose that the transgene "is either causative of the loss of fin phenotype or tightly genetically linked". This implies that the transgene disrupts (or is positioned close to) a genetic regulatory element that is necessary for fin positioning or fin induction.
This is an extremely interesting implication of the mechanism affected in the mutant that you cannot ignore. I suggest, you produce a crispr/CAS deletion of the non-coding region around the insert site of a wild-type chr17:46,219,972 (see next comment) to demonstrate lack of the dorsal fin in a second genetic allele at this site (a deletion allele). This may be a one-generation experiment with sufficiently high gRNA concentration as a proof of principle experiment for the matter of this manuscript.
Your response: This is an ¬excellent suggestion. However, the smb insertion locus is very repetitive, and difficult to PCR amplify, let alone design gRNA to specifically mutagenize the region. Nevertheless, per this reviewer’s suggestion we designed 6 gRNAs to the best of our ability and injected them. We did not observe any fin loss in these injected mutants, but we cannot be sure we are effectively mutagenizing the locus since PCR amplification was difficult. Therefore, we have not included the negative results from this experiment in this manuscript, yet we will continue to address this important question going forward and hope to include this experiment in future work.
Reviewer2 reply: Unfortunate, the suggested experiment doesn’t seem to be within the scope of a revision.
We thank the reviewer for their initial suggestion and their understanding of the technical difficulties of this experiment.

p.8 The smb-causing transgene drives Gal4 in ectopic tissues and does not disrupt a coding sequence. 2nd paragraph: "we crossed a smb mutant to UAS:E2Crimson fish to visualize Gal4 activity": pharyngeal arch labelling: Fig.S2b clearly shows labelling of the hyosymplectic and the ceratohyal, the two elements of the hyoid arch, to a knowledgeable observer. All others may not understand the significance of the image. If you could provide a higher power view of the image, that shows the round structure of the cartilage cells within these red elements, the figure would convince readers that head cartilages are stained.
Your response: We have provided a higher power view of the craniofacial cartilage elements in smb(sox10:gal4) fish including new data colocalizing expression with sox9:EGFP to definitively show these are chondrocytes/cartilage in the head (Fig. S2). We also include text to clarify the importance of this result (lines 191-193).

Reviewer2 reply: The supplementary Fig. 2B is beautiful and clear. Please include in your figure legend and in the text (line192) that all elements of the first arch are visible: Meckel’s cartilage, hyosymplectic and ceratohyal and don’t leave out one or the other in your figure legend and text.
We labeled the cartilage elements that the reviewer mentions here in the figure, figure legend, and text. We note that the reviewer is incorrect in their assertion that these are all derivatives of the first arch, the hyosymplectic and ceratohyal cartilages are both derived from the second pharyngeal arch.

Please don’t show Fig.S2C. The notochord staining of the sox9aEGFP transgenic line is not helpful and appears to be a peculiarity of this line. You also don’t even mention it in the text nor in the figure legend. Please instead include Fig.S2 B form version1 (embryo tail only!) because it shows neural tube staining and fin mesenchymal staining much better than the new Fig.S2C.
We changed this as suggested.

© 2024. Published by The Company of Biologists under the terms of the Creative Commons Attribution License (https://creativecommons.org/licenses/by/4.0/).

I'm sorry that I have to confess that I don't understand which tissue is stained in Fig. S2D. I'd expect to see somites/myotomes at 5dpf in the paraxial mesoderm but the tissue is not segmented. Is this non-migrated sclerotome? Please add an explanation to the legend of S2.
This reviewer is confused about the stage in this figure which was clearly indicated in both the text and the figure panel in the last submission. This is 5.1 mm standard length stage, not 5 dpf. Nevertheless, in an effort to avoid other readers from missing the stage labelled in this figure, we now additionally include explicit language in the figure legend indicating the stage, as well as calling the specific panel in the text where we state the stage.

Figure legend S2C: "Caret indicates ectopic labeling of caudal fin fold fibroblasts by sox10:Gal4;UAS:E2Crimson.
Cancel "ectopic"! You just document the expression of your transgenic line as it is. The mesoderm expression is the topic of the paper anyway.
Good point, we corrected this and thank the reviewer for their comment.

Fig.S2 a&b,c,d: staining in the paraxial mesoderm and mesenchyme of the median fin fold: instead of saying "We conclude that the smb phenotype is genomic integration site-dependent and is not strictly due to Gal4 protein expression in fin mesenchyme. These results motivate the hypothesis that transgene integration into a specific genomic locus affects endogenous gene expression or gene function or both to cause the smb phenotype." You should explicitly state in the text that aside from the labelling of the hyoid arch cartilages, the transgene provides two functions: 1. It can act as an enhancer trap, staining mesodermal cells in the trunk and median fins and 2. at the smb genomic integration site, it mutates a dorsal-fin specific regulatory element which leads to the smb phenotype (hence the above suggestion of creating a second allele by crispr/CAS deletion, to verify this assertion)
Your response: This is a good comment and this section requires clarification which we provide here and in the text. We do not believe that the mesodermal labeling in smb is strictly due to enhancer trap activity since different (non smb) integrations also label these cells. Although we cannot conclude that these new insertions aren't also trapping other mesodermal enhancers, it seems more likely that the shortened sox10 regulatory sequences in the construct drive mesodermal expression. We clarify in the text that this version of sox10 regulatory sequences is shorter than those previously reported likely accounting for the mesodermal expression (see above).
Reviewer2 reply: I agree with your interpretation. Your argument of 3 independent transgenic lines showing the same mesodermal expression is convincing. Line 208 replace "presumably" with "likely". Line 212 replace "other" with "in our".
Lines 215-216 proposal: These results motivate the hypothesis that transgene integration specifically into the smb genomic locus …
We made changes to the statements recommended by the reviewer for clarification.
p.9/10 The dorsal fin skeleton fails to develop and anal fin skeletal elements are disorganized in smoothback
p.10 second paragraph: the sox9a:EGFP-line labels cartilaginous elements, i.e. the endoskeletal radials of the dorsal fin. However, you need to show that, at an earlier stage, the dorsal fin bud/field mesenchyme does not accumulate at its regular position above somites 11 – 15, i.e. if the mesenchyme fails to accumulate, there is no sign or fin induction! You can visualize this by "Nomarsky" optics of the dorsal fin fold of 2 weeks old fish. Bleach pigment if it is in you way or use a pigment mutant so that you can see the initial undifferentiated "bud" mesenchyme. The question is how a lack of fin mesenchyme aggregation comes about. In any case, it is central to the interpretation of the mechanism causing the smb phenotype, viz. axial patterning/fin induction vs. a late failure of fin bud growth/differentiation. It is important that you report evidence that favors one vs. the other cause of the smb
Your response: Per this reviewer's request, we now include new DIC/Nomarski images of wild-type and smb fish at stages of fin induction. Together, with the fli1:EGFP data these results strongly favor the developmental mechanism is due to a lack of fin induction and not growth or differentiation (Fig. 3). Further, in smb mutants with a partial dorsal fin (Fig. 1), the cells that do reach the site of future fin development are able to undergo the skeletogenic differentiation program suggesting all the signals for this differentiation are unaffected in smb
Reviewer2 reply: Fig.3 is convincing. The lack of mesenchymal accumulation is shown well, also in the fli1:EGFP transgenes.
We thank the reviewer for their suggestion which bolsters our results and interpretations.

© 2024. Published by The Company of Biologists under the terms of the Creative Commons Attribution License (https://creativecommons.org/licenses/by/4.0/).

Text: line 283: replace “look at” by “view of”. Line 285: replace “ectomesenchyme” by “mesenchymal”: It’s true that fli1:EGFP labes ectomesenchymal i.e. crest derived cartilage progenitors. But this is not what you wish to imply wih the median fins! You show by lineage labelling the fin mesenchymal cells are sclerotome-derived. Who knows why the fli-line also labels fin mesenchyme? Just avoid the unnecessary theory-laden “ecto”mesenchyme.
Thank you for this good suggestion. We agree and made the edit as recommended.

Line 291: the earliest reported step in median fin formation, potentially induction of fin bud mesenchymal progenitors…..The sclerotomal progenitors might be induced but fail to migrate, hence I suggest to downscale
As suggested we include the word potentially in this sentence to downscale our hypothesis.

Line303: hand2:EGPF labels the posteriormost rays … give a reference
We’re unclear on this suggestion. On line 303 (now line 304) where we stated that *hand2:EGFP* is restricted to the posterior rays we referenced Nachtrab since that manuscript contains the first observation of the restricted fin expression of that transgene. On line 309 we further their analyses by quantifying and comparing the number of rays expressing the transgene between wild types and *smb* heterozygous mutants, this is a finding from our work not requiring a citation.

p. 9/10 in comparison to p.6, introduction: in your introduction you introduce the t-box gene eomesa as a necessary factor for dorsal fin formation in zebrafish and carp. Even though this gene was found to be necessary for dorsal fin develoment, its expression pattern has not been reported. Two other t-box transcription factors, tbx5 and tbx4 are necessary for paired fin/limb formation. They are the earliest markers of pectoral and pelvic fin formation in zebrafish, respectively.
Is eomesa likewise an early marker of the dorsal fin mesenchyme? It would be extremely rewarding if you could undertake this in-situ hybridization, because it would demonstrate the activity of another t-box gene during early fin development and would add to the (ancient) homology argument of unpaired and paired fins the developmental perspective of t-boxes regulating the developmental induction of all appendages!
In addition, you could argue that smb fish don't show early signs of dorsal fin induction instead of arguing smb don't show cartilage radial differentiation in the fin mesenchyme, which is too late.
Your response: This is an excellent idea. In fact, we have an active project examining eomesa expression and function ongoing in the laboratory. Within this separate study, we performed HCR in situ as this reviewer suggests and observed that eomesa is indeed expressed in the dorsal and anal fin bud of wild types! These exciting data suggest that eomesa may be a t-box median fin gene as this reviewer speculates and have important implications for the (ancient) unpaired and paired fin homology argument. These results are central to the thesis work of another graduate student and we plan to reserve these findings and their implications for a separate study, but share them here for the reviewer to observe (see below). I hope that this same reviewer agrees to review this upcomingmanuscript as well!

p11/12: smb mutants do not establish the dorsal fin bud
p12 first paragraph: "These results motivate the hypothesis that smb affects one of the earliest reported steps in median fin formation":
As pointed out above, you should be able to show that "the earliest steps in median fin formation" is affected in smb mutants and not leave it to a hypothesis, open to discussion.
We have added DIC images of smb and wt fish at fin induction stages. We also present data in the manuscript where we show no dorsal fin bud/mesenchyme using a transgenic line (fli1a:EGFP) that strongly labels the fin buds in wild types and reveals that they are absent in mutants (Fig. 3).
Reviewer2 reply: Thank you for sharing the interesting eomesa in-situs. I’m surprised that the domain appears restricted to the posterior part of the fin anlagen. Else, see my response above.
The hypothesis that “smb disrupts the earliest reported steps in median fin formation” is supported by the demonstration of lack of the mesenchymal anlagen.
We were also surprised, and excited, by this expression pattern and look forward to results from our ongoing *eomesa* study.

p.13 The sclerotome is the source of the median fin skeleton this is a strong section in the manuscript! May I encourage you to provide a higher magnification image of Fig.4B such that the reader can actually appreciate the condrocytic distal and proximal radials at the ends of the

© 2024. Published by The Company of Biologists under the terms of the Creative Commons Attribution License (https://creativecommons.org/licenses/by/4.0/).

arrowheads. (You might have to cut away the muscles) There is only little mention in the literature that the endoskeletal radials are of sclerotomal origin, though they were always assumed to be. The neural crest hypothesis also only explicitly addressed the origin of the lepidotichs. Your lineage tracing image could show that both skeletal compartments originate from one progenitor population.
Your response: This is a good suggestion. We added zoomed in images of the proximal and distal radial chondrocytes clearly demonstrating Cre mediated recombination in these cells by the nkx3.1 driver (Fig. S6). We also clarify in the discussion that the sclerotome is the source of both radials and rays (lines 456-458).
Reviewer2 reply: Good and clear images and good conclusions
Thank you. We agree these images better support our claims.

p14: Sclerotome expansion is decreased in smb mutants please explain what we see in Fig.5 There is a paraxial region of expression lateral to the vertebral column? but there also is a detached "dorsal expression domain". Are these cells the ones that give rise to the dorsal fin? Which pax9 cells did you count in your quantification?
Your response: The population of the sclerotome lateral to the vertebral column is known as the “notochord-associated sclerotome” (Ma et al. 2018) and is initially part of the ventral sclerotome region. We now clarify this in the main text (lines 355- 356) and cite appropriate sources. We have not focally mapped the dorsal expression domain and what structures it gives rise to in the larval/adult body. These experiments are active in the lab and we hypothesize that the dorsal domain gives rise to the dorsal fin but we have not yet demonstrated this definitively. However, we have added a section hypothesizing this in the discussion (lines 459-474). For the pax9 volume analysis, in the original version of the manuscript we quantified the dorsal and ventral domains (which include the notochord-associated cells) together for the 5 somites immediately posterior and anterior to the vent. We now also include separate analyses quantifying the dorsal and ventral domains of the posterior trunk independently at 24 and 26 hpf and find a significant decrease in both at 24 hpf but only in the dorsal but not the ventral domain at 26 hpf (Fig. S7-9). We clearly describe what domains were quantified in each analysis. Thank you for this suggestion that improved the manuscript.
Reviewer2 reply: Thank you for more precisely describing the pax9 domains and for the quantification

Your quantification of 24hpf and 26hpf pax9 domains give support to your depletion model (line 424 ff.) if we assume that the measurable depletion between 24-26 hpf leads to a smaller precursor population that cannot be compensated for at later stages. The idea is valid, however, you argue yourself that by 30hpf, the reduction of the sclerotome is not measurable any more.

Your quantification of 24hpf and 26hpf pax9 domains give support to your depletion model (line 424 ff.) if we assume that the measurable depletion between 24-26 hpf leads to a smaller precursor population that cannot be compensated for at later stages. The idea is valid, however, you argue yourself that by 30hpf, the reduction of the sclerotome is not measurable any more.

NOTE: We have removed unpublished data that had been provided for the referees in confidence.

We note that this stage was not included in the original submission. We added these data because this reviewer asked for them. We did not include these results in the initial submission because the assay is no longer marking just sclerotome, see lines of evidence below indicating that this is no longer a specific sclerotome marker at this stage.

In Fig S9, much of the structures are no longer considered sclerotome, but sclerotome-derived cells which are becoming differentiated (Ma. 2018). This is evidenced by the tenocytes visible between the somite segments. Further, in the same paper that first describes the zebrafish bipartite sclerotome they define *two* regions of the sclerotome and explicitly state that after the ventral cells begin to migrate they are “sclerotome-derived cells” and no longer sclerotome. Thus, we are not actually measuring just sclerotome per se in this assay because we are not able to discern the individual domains for quantification. We note that the corresponding author from Ma et al. is a coauthor on our manuscript.

© 2024. Published by The Company of Biologists under the terms of the Creative Commons Attribution License (https://creativecommons.org/licenses/by/4.0/).

Further, *pax9* is beginning to turn down in an A-P wave at these stages confounding interpretation. See our attached unpublished developmental series of *pax9* expression in the trunk where *pax9* expression is no longer in discrete domains at 36 hpf before turning down by 44 hpf.

In summary, *pax9* expression is no longer a sclerotome specific marker. We are keeping the 30 hpf *pax9* expression data in the manuscript (in the supplement) per this reviewer's initial request, but indicate in the text that it is no longer a sclerotome specific marker.

Fig.S9: the smb image exhiits a differently sized pax9 domain than the wt at 30hpf, which I would have expected according to your analysis at 24 and 26hpf. I realize, there are expressivity differences between individual embryos but this one doesn't show that wt and smb have identical amounts of pax9 expressing precursors. The smb embryo you show has clearly weaker pax9 expression than the wt, does it even have segmentation problems (?) as it doesn't show the chevron shapes of the wt-somites. It is difficult to acknowledge that the pax9 domains are alike in smb and wt at 30hpf. Are they really?

We unequivocally stand behind our rigorous quantification of the 30 hpf time point which was done identically to the other time points. Because we include the individual points in the plot, you can see the variation among *smb* heterozygous mutant individuals. The statistics are very conclusive that there is no significant difference between the samples at this stage, although we are not strictly measuring sclerotome at this stage (see argument above).

If so, this is a problem, because the depletion-model relies on the lack of precursors. Have you tried to use a different measure to quantify your precursor pool, e.g. EdU? It would be easier to my mind to accept an early embryonic depletion that leads to lack of material at later larval developmental stages thus affecting the unpaired fins rather than observing an only transient retardation of precursor development that is compensated for only 4 hours later by 30hpf.

To clarify, at this stage *pax9* is not strictly labelling the sclerotome, it's labelling structures like the syndotome which is unchanged in *smb* mutants (see fig 6). We do not interpret these data to indicate that there is compensation.

Perhaps I am missing an argument here but I suggest you revisit the 30hpf results and perhaps apply a more robust method of quantification.

This is a robust method of quantification of the HCR signal. Again, the same method did find differences earlier, at stages when *pax9* is specifically marking the progenitors, indicating that the assay is sensitive enough to uncover differences. However, as discussed above, at the 30 hpf stage *pax9* does not strictly mark the sclerotome and we can no longer discern discrete domains.

We now clarify in the main text the caveats associated with using *pax9* at this stage.

p15/10: Early sclerotome derivatives remain unaffected in the smb mutant
You conclude that "that derivatives of the sclerotome that emerge at early larval stages are unaffected in the smb mutant, while the later forming structures like the median fins are more sensitive".
In this context, it may be interesting to probe for an anterior-posterior axial patterning defect in the smb mutant while mesoderm segmentation per se might be correct. In the paper of Frank van der Hoeven et al. 1995. Mechanisms of Development 54 9-21. the authors point out that the mesodermal axial expression patterns of hoxd genes show the expected collinear expression. Especially hoxd11/hoxd12 show an anterior border of their expression domains at somite 11 and 17, respectively, which marks the region of the anterior margin of dorsal and anal fins. Again, an in situ hybridization with these markers around 22hpf should show if smb shows an a/p patterning defect that would consequently lead to a lack of dorsal fin induction vs. a fin induction defect where smb fish would show the regular pattern of paraxial hox gene expression but only later lack dorsal fin mesenchyme formation and consequently eomes expression.
Your response: This is an interesting idea. We performed this experiment as suggested and found that hoxd expression was not affected in smb. We added these new data to the manuscript (Fig. S5) and discuss that the smb insertion likely directly affects fin induction rather than affecting hox gene expression which secondarily affects fins (lines 313-326).

Reviewer2 reply: Line 323-326: Rephrase your conclusion from the hoxd12 expression pattern or maybe put it into a different context, see last paragraph. Here the lengthy reasoning:

© 2024. Published by The Company of Biologists under the terms of the Creative Commons Attribution License (https://creativecommons.org/licenses/by/4.0/).

Even though “fin induction” is a pre-molecular term describing a process that leads to aggregation of the fin mesenchyme as the earliest visible read out, this process is generally thought to include a “positioning” component. This “positioning” has been proposed to be dependent on the borders between axial “segments”, that can be visualized by the collinear expression of hox genes. Thus, if you find that the expression pattern of a hox gene is not disturbed in smb mutants, this actually argues in favor of a normal patterning process and consequently not in favor of disruption of limb induction due to confounded identities of the relevant somites. The “induction” defect that leads to lack of a mesenchymal aggregation must thus develop from a defect other than axial pattern identity issues.

You have shown the lack of a mesenchymal condensation in smb-transgenics. Thus, there is a process blocked at an early stage of fin development. Showing correct axial expression of hoxd12 alone may show that axial patterning is not disrupted and therefore this component of “fin induction” in not affected (though expression of a single hox gene is perhaps not sufficient for such a conclusion.)

Your observation is ,however, in favor of your hypothesis that the lack of dorsal fin mesenchyme is a problem of “sclerotme expansion” thus strengthening your view, that smb controls precursor supply. You may more easily discuss your hoxd12 results in this context.

Thank you for your suggestion. We now include some additional language to ensure that we encompass the arguments that the reviewer makes here.

***** Reviewer 3 Advance Summary and Potential Significance to Field:
This reviewer was enthusiastic about the manuscript after first review and finds the manuscript additionally improved after revision. The manuscript addresses an important gap in the literature, the timing and origin of the precursors that give rise to the median fins (dorsal and anal fin), by characterizing a novel dominant phenotype caused by a transgenic insertion. The manuscript is well-written and the conclusions and model are well-supported by the data shown.

Reviewer 3 Comments for the Author:

The authors should read the ZFIN Nomenclature Conventions (https://zfin.atlassian.net/wiki/spaces/general/pages/1818394635/ZFIN+Zebrafish+Nomenclature+Conventions), Section 4.3 on Transgenic lines and constructs, and specifically 4.3.3 on Transgenic lines, where it states “Transgenic lines are of two types, those that are known to create alleles of genes and those that are not known to create alleles of genes”. Authors should use the appropriate convention for the latter. Using these guidelines, they should assign an allele designation for the insertion allele and clearly define this designation in the Methods section (they cite allele designations for all other lines they use in the paper in the methods, but not their own). They do assign an allele designation on Line 143, but it is not consistent with ZFIN nomenclature guidelines. Since they only have one smb insertion allele, it’s fine to state the zygosity (and not the specific allele designation) every time, once it has been initially defined.

We agree that nomenclature is important. We read the ZFIN Nomenclature Conventions very carefully before choosing how to name this unconventional allele. Further, to be certain we used proper nomenclature, we directly contacted the ZFIN nomenclature coordinator before the original submission. For reference we include a screenshot of the correspondence from 3/19/24. We elected to adhere to the instructions direct from the ZFIN nomenclature committee below. This reviewer makes a good point that we need to assign an allele designation in the Methods section, which we now include.

Examples of where terminology is confusing in the text (may not include all examples; authors are encouraged to carefully review for other instances in the text, figures, and legends):
•Line 127: clarify by saying “dominant mutant”
•Lines 132, 136, 186, 274, 294, 297, 300, 302, 304, 307, 358, 362, 368, 369, 406, 420 : zygosity is not stated
•Line 137: “depletion in smb mutants”

© 2024. Published by The Company of Biologists under the terms of the Creative Commons Attribution License (https://creativecommons.org/licenses/by/4.0/).

•Line 169: "to determine whether homozygosity for the smb transgene" – if there allele were defined in the section above, this would streamline and clarify the description here, e.g. "to determine whether homozygosity for the smb transgenic insertion allele, smbco3021Tg, …"

In this instance, we do not include the full allele designation because we have already defined it above and in the methods. Moreover, this adheres to the suggestion on nomenclature usage in the above email from Amy Singer to only define at the first usage.

•Line 187: clarify what "double transgenic fish" means. Here you are "using" smb for the transgene thatcaused the insertion allele, correct? This may be confusing to a reader.

This is a very important suggestion. It is difficult to know how to handle nomenclature when referring to the mutant phenotype versus the transgene expression patterns which are both due to the same genomic feature. After much debate and consideration, we now clearly state that when referring to the mutant phenotype we say *smb* mutant, including the zygosity. In contrast, when describing the expression patterns driven from the transgene we use *smb* (*sox10:Gal4*). We include *smb* before the transgene name to discern it from other *sox10:Gal4* constructs and integrations. This usage is only relevant to Figure S2. We hope that this reviewer, and all our readers, find this usage easy to understand.

•Line 263: "two dominant mutants". Mutations, alleles, and phenotypes are dominant. Just stating the genotypes of the parents would be clearer.
•Line 266-8: In this section (line 266-268, it would be easier if the authors just clearly and consistently stated the genotypes of the phenotypic classes. They do state the lof zygosity, but not smb zygosity, in the Figure associated with this text.
•Line 275: "smb defect" is unclear
•Line 289: "stage-matched smb", zyogosity and mutant not stated
•Line 290: "smb disrupts" implies there is a wild-type smb that functions to disrupt a process
•Line 309: term "partial mutants" is unclear
•Line 411: "less sensitive to smb" – the meaning here is unclear.

We made all these changes, and others, after carefully reviewing the entire manuscript for other issues. Thank you for carefully reading our manuscript and identifying where the terminology remains unclear.

<u>Third decision letter</u>

MS ID#: DEVELOP/2024/203025

MS TITLE: The sclerotome is the source of the dorsal and anal fin skeleton and its expansion is required for median fin development

AUTHORS: Raisa Bailon-Zambrano, Margaret K. Keating, Emily C. Sales, Abigail R. Nichols, Grace E. Gustafson, Colette A Hopkins, Katrinka M. Kocha, Peng Huang, Lindsey Barske, and James T. Nichols

I am happy to tell you that your manuscript has been accepted for publication in Development, pending our standard publication integrity checks.

© 2024. Published by The Company of Biologists under the terms of the Creative Commons Attribution License (https://creativecommons.org/licenses/by/4.0/).
